# Recovering Drug-Induced Apoptosis Subnetwork from Connectivity Map Data

**DOI:** 10.1155/2015/708563

**Published:** 2015-03-25

**Authors:** Jiyang Yu, Preeti Putcha, Jose M. Silva

**Affiliations:** ^1^Department of Precision Medicine, Oncology Research Unit, Pfizer Inc., Pearl River, NY 10965, USA; ^2^Department of Pathology, Columbia University, New York, NY 10032, USA; ^3^Department of Pathology, Icahn School of Medicine at Mount Sinai, New York, NY 10029, USA

## Abstract

The Connectivity Map (CMAP) project profiled human cancer cell lines exposed to a library of anticancer compounds with the goal of connecting cancer with underlying genes and potential treatments. Since the therapeutic goal of most anticancer drugs is to induce tumor-selective apoptosis, it is critical to understand the specific cell death pathways triggered by drugs. This can help to better understand the mechanism of how cancer cells respond to chemical stimulations and improve the treatment of human tumors. In this study, using CMAP microarray data from breast cancer cell line MCF7, we applied a Gaussian Bayesian network modeling approach and identified apoptosis as a major drug-induced cellular-pathway. We then focused on 13 apoptotic genes that showed significant differential expression across all drug-perturbed samples to reconstruct the apoptosis network. In our predicted subnetwork, 9 out of 15 high-confidence interactions were validated in the literature, and our inferred network captured two major cell death pathways by identifying BCL2L11 and PMAIP1 as key interacting players for the intrinsic apoptosis pathway and TAXBP1 and TNFAIP3 for the extrinsic apoptosis pathway. Our inferred apoptosis network also suggested the role of BCL2L11 and TNFAIP3 as “gateway” genes in the drug-induced intrinsic and extrinsic apoptosis pathways.

## 1. Introduction

One goal of biomedical research is to better understand human diseases such as cancer by studying gene patterns associated with diseases and using them to find the best potential treatments. Recently, Todd Golub and his colleagues at the Broad Institute initialized the “Connectivity Map” (CMAP) project [1, 2] to make these disease-gene-drug connections by utilizing microarray technology. High-throughput microarrays are able to profile gene expression at a whole-genome level and can be used to detect signatures under certain perturbations or phenotypes in cells [3]. Since the therapeutic goal of most anticancer drugs is to induce tumor-selective cell death [4], it is reasonable to hypothesize that apoptosis may be a major cellular mechanism targeted by anticancer drugs. It is therefore critical to understand the specific cell death pathways that are activated by drugs. This would help to better understand the mechanism of how cancer cells respond to chemical stimulations and improve the treatment of aggressive human tumors. Because the CMAP database contains profiles from a large collection of human cancer cell lines containing information on how cells respond to chemical stimulations, it can be used to test the hypothesis that the apoptosis pathway might be a major responsive program to drug perturbations in cancer cells. One can do this by enrichment analysis of apoptotic genes in drug-responsive genes or in differentially expressed genes in drug-exposed cancer cells [5–10]. CMAP data also contains dynamic transcriptional activities of most genes across diverse conditions, giving sufficient data for associating the activities of genes of interest with each other and for reconstructing parts of the apoptosis pathway in the context of drug-exposed cancer cells. In this study, we used CMAP gene expression profiles to test the hypothesis that apoptosis may be a major drug-induced cellular mechanism. We then employed a Gaussian Bayesian network modeling approach to reconstruct the subnetwork of the drug-induced cell death pathway. To minimize the effects of heterogeneity from different tumor types, our study focused on a single breast cancer cell line, MCF7.

The death of mammalian cells is induced by intracellular cysteine proteases known as caspases. Caspases are first synthesized as largely inactive zymogens known as procaspases and are later activated through posttranslational mechanisms. Two principal pathways of caspase activation have been recognized [11–13]. One pathway, which is of more ancient origin and evolutionarily conserved, is known as the stress pathway, mitochondrial pathway, or intrinsic pathway. It is induced by developmental cues and diverse intracellular stresses. This pathway begins with the activation of caspase-9 on a scaffold formed by Apaf-1 in response to cytochrome c release from damaged mitochondria. It is known to be regulated primarily by proteins from the Bcl-2 family. The other pathway is known as the extrinsic pathway and is triggered by the so-called “death receptors” on the cell surface. The death receptors are engaged by cognate ligands of the tumor necrosis factor (TNF) family. This pathway begins with the activation of caspase-8 (and caspase-10 in human cells), via adaptor proteins including Fas-associated death domain protein (FADD). Once activated, caspase-9 in the intrinsic pathway or caspase-8 (-10) in the extrinsic pathway activates downstream “effector caspases” including caspase-3, caspase-6, and caspase-7. In an expanding cascade, these caspases carry out the execution phase of cell death. To better understand whether anticancer drugs target the intrinsic and extrinsic apoptosis pathways and identify specific pathways or interactions activated by anticancer drugs, we crossed our predicted drug-triggered apoptosis network with literature-validated interactions. We were able to identify key players as well as interactions in the drug-induced intrinsic and extrinsic pathways. Our results shed light on the mechanism of action of drugs in cancer cells and may lead to improved treatments that target key apoptotic proteins that are most related to drug response.

## 2. Data

### 2.1. CMAP Dataset

The CMAP “build 02” gene expression dataset (http://www.broad.mit.edu/cmap/) contains over 7,000 profiles of cancer cells that have been exposed to perturbations by 1,309 compounds and contains data from five human cancer cell lines: MCF7, PC3, SKMEL5, HL60, and ssMCF7. The microarray platforms used include Affymetrix HT_HG-U133A and HT_HG-U133A_EA. To avoid the effects of tumor heterogeneity and multiple microarray platforms, without loss of generality, we only focused on samples from the breast cancer cell line MCF7 that were profiled using the Affymetrix HT_HG-U133A platform. The dataset is composed of 404 control and 2,417 compound-perturbed samples. The HT_HG-U133A microarray platform contains 22,268 Affymetrix probe sets representing 13,262 genes. The GCRMA method [14] was used to normalize the data.

## 3. Results

### 3.1. Drug-Responsive Signature Analysis

**Figure 1:**
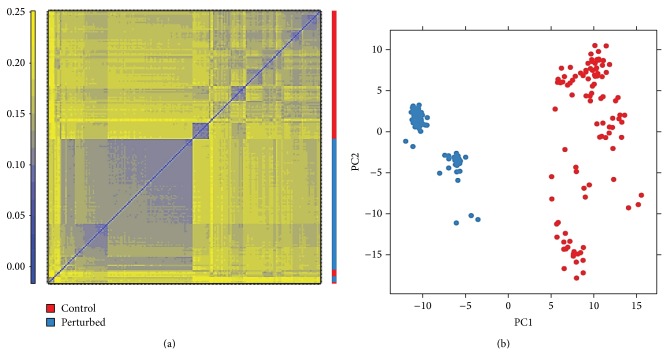
Heat map and PCA plots of drug-perturbed profiles in CMAP of 22,277 informative probe sets. Nonvariant probes across all samples are filtered out by IQR < 0.5. The heat map of distances (a) and the PCA plot (b) between profiles of CMAP data including randomly selected 100 control and 100 drug-perturbed samples.

To identify drug-responsive signature genes at a transcriptional level in cancer cells, one approach is to perform differential gene expression analysis by comparing drug-perturbed samples with controls. However, since the dataset contains samples tested with over 1,000 chemical perturbations, it is important that we take into account the diverse mechanisms of actions of the different compounds. One solution would be to perform differential expression analysis for each compound separately and then combine the results together using a *P* value-based Fisher's method or Stouffer's *z*-score approach to obtain the overall differential expression level for each gene across all compounds. However, a limitation with this type of analysis has to do with the fact that each compound only has a limited number of perturbed samples and even smaller number of control samples. This would cause the statistical power to be extremely low for individual compound analysis and would result in an inaccurate estimation of parameters and a high false positive rate. Additionally, another known issue with this type of “Separate-then-Combine” analysis is a low precision rate, which means there is a high occurrence of false positives among the most differentially expressed genes or top-hits. One way to overcome this drawback is to combine all compounds together at the beginning, as known as a “complete pooling” method. Although different drugs may have distinct mechanisms of action and different target proteins, it may still be reasonable to group them together. One reason is that there are a relatively limited number of pathways or mechanisms through which cells respond to chemical stimulations. Also, compounds tested for cancer treatment are known to share some common characteristics. For example, a large number of anticancer drugs are known to induce cell death or repress cell growth programs. In addition, the combination or “complete pooling” strategy increases the sample size from less than 5 to thousands, dramatically increasing the statistical power for inferring true responsive genes across all compounds. This assumption is also confirmed by the fact that most perturbed profiles are clustered together as shown in Figure 1. These results indicate that the variability of transcriptional profile for the same type of cell (MCF7 in this study) due to drug heterogeneity is much smaller than that caused by different chemical stimulations.

To estimate the effect of each compound on gene expression and to test the significance of differential expression for each probe set, we used a linear modeling method with empirical Bayes moderated *t*-test [15]. A nonparametric Bonferroni procedure was employed for multiple comparison correction. Using a false-discovery-rate (FDR) threshold of 0.05, we identified 137 upregulated and 90 downregulated probe sets, representing 112 overexpressed and 79 underexpressed genes, respectively (Table S1, available online at http://dx.doi.org/10.1155/2015/708563), in drug-perturbed cancer cells.

### 3.2. Enrichment of Apoptosis Pathway

**Figure 2:**
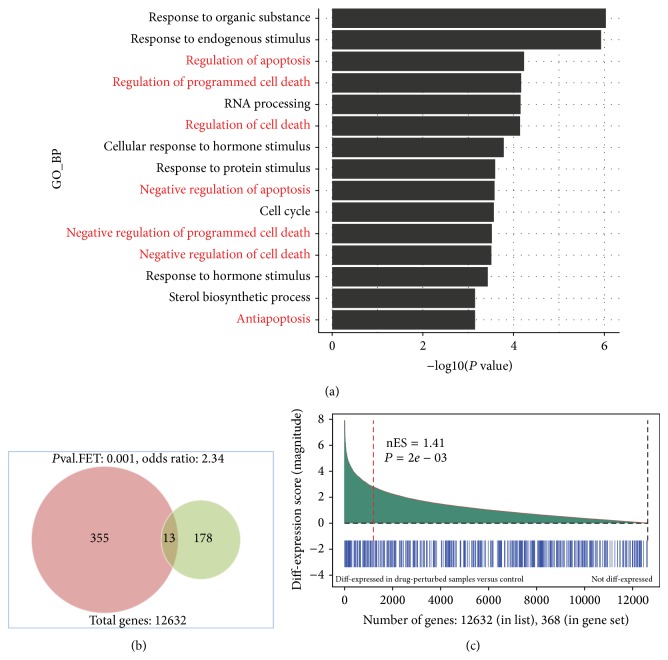
(a) Top enriched (*P*_value_ < 0.001, FDR < 0.1) GO BP terms by top differentially expressed genes (FDR < 0.05) with apoptosis-related processes highlighted in red; summary of (b) Fisher's Exact Test and (c) Gene Set Enrichment Analysis (GSEA) to test whether apoptosis pathway with 368 apoptotic genes is enriched in drug-induced signature genes. For GSEA method, absolute mean was used to summarize the enrichment and 10,000 gene permutations were used to produce the significant level.

As described previously, one of the most important mechanisms induced by oncotherapeutics is cell death programs. More specifically, we hypothesized that the apoptosis pathway may be a major drug-induced program. Enrichment analysis was proposed to validate this hypothesis. Indeed functional enrichment analysis by DAVID [16] confirmed that apoptosis or cell death pathway is a major biological process triggered by anticancer compounds as half of top enriched GO BP terms (FDR < 0.1, *P* < 0.001) by drug-responsive signature genes are associated with apoptosis (Figure 2(a)). Furthermore, by searching the Gene Ontology database [17], we obtained a list of 380 human genes that were annotated with apoptosis-related GO terms (Table S2). 211 genes were annotated as proapoptotic by induction of apoptosis, positive regulation of apoptosis, and negative regulation of antiapoptosis. 194 genes were annotated as antiapoptotic by negative regulation of apoptosis and positive regulation of antiapoptosis. 25 genes were involved in both positive and negative regulation of apoptosis. We then performed enrichment analysis with differentially expressed genes of drug-perturbation in the apoptosis pathway. Two methods were employed to do this analysis: the first method was Fisher's exact test to validate whether known apoptotic genes were overrepresented in a selected differentially expressed drug-responsive gene set. The second method was to test the known apoptotic genes using Gene Set Enrichment Analysis (GSEA), which does not perform a selection on differentially expressed genes, but instead it considers the entire set of genes and their differential expression as the background. For Fisher's exact test, a set of previously identified 191 signature genes with a threshold of FDR < 0.05 and all 12,632 genes in the microarray were used to fit the null hypergeometric distribution. For GSEA, the mean of absolute value of differential expression was used as enrichment score because apoptotic genes could be either up- or downregulated in drug-perturbed samples. The significance of the enrichment scores was tested against 10,000 permutations of gene names.

There are 13 genes (Table 1) that overlap between the 191 drug-inducement signature genes and the 368 human apoptotic genes in our dataset. The significance level of Fisher's exact test for this overlap is approximately 0.001 (Figure 2(b)), consistent with the result from GSEA, which had a *P* value of 0.002 (Figure 2(c)). Therefore, both methods confirm that the preidentified drug-induced signature genes are significantly enriched in the human apoptosis pathway. In other words, we were able to validate our hypothesis that the apoptosis pathway is a major cellular mechanism targeted by anticancer drugs. Furthermore, separate analysis of pro- or antiapoptotic genes (Figures S1-S2) showed that drug-responsive genes were enriched in both positively or negatively regulated apoptosis gene sets. Since the analysis was done using the combination or “complete pooling” strategy, the significance of these results suggests that 13 drug-induced apoptotic genes in our gene set are responsible for a highly conserved response to multiple chemical compounds in the context of breast cancer.

### 3.3. Bayesian Network

We next asked the question of how the 13 identified genes work together systematically and whether we can recover the underlying network structure of their interactions. This would help us to better understand the mechanism of how cancer drugs induce the apoptosis pathway at a global systems level. In order to infer the underlying signaling and transcriptional and causality network of the 13 drug-induced apoptotic genes, we used one of the best methods for network reconstruction in the literature, the Bayesian Network or Graphical Model [18–22]. The details of the method are described below.

#### 3.3.1. Data Modeling

A Bayesian network represents the dependence structure of a joint probability distribution of multiple variables, which can be factorized into a product of distributions of each individual node conditioning on its parents. To model the local distribution of each node conditioned on its parents, a commonly used method for continuous data is to discretize data points into bins and then fit a multinomial distribution to the discretized data. However, data discretization results in a loss of information and can be highly sensitive to the number of bins the data is split into. Furthermore, due to the continuous nature of microarray data and the marginal normality of many genes in this study as shown in Figure 4, we determined it would be more accurate to employ a continuous model. We therefore used a conditional linear Gaussian model [23] for the local distribution of each node as shown below:

(1) gi ∣ parentsgi,β,αi,σi2,G ~N∑jβi∗parentjgi+αi,σi2.

This model can be recognized as a linear regression model, in which node *g*_*i*_ is the response variable, its parents are covariates, and the noise follows a white Gaussian distribution with mean 0 and variance *σ*_*i*_^2^.

#### 3.3.2. Parameter Learning

Given the linear regression model for the local distribution, a classical Maximum Likelihood or Least Squares approach can be used to estimate its parameters. However, various studies in statistics have suggested that Bayesian approaches or Bayes estimators are more robust than a Frequentist maximum likelihood method [22], especially when the sample size is small or the data is noisy. Therefore a Markov Chain Monte Carlo (MCMC) simulation-based Bayesian computing method was used to estimate parameters of the model. To select the priors for the Bayesian model, two principles were followed: one is conjugation for computing easily as the posterior will fall in the same distribution family as prior, and in our case, the prior would be Gaussian for conditional coefficient and Inv-Gamma for variance; the other is global and local parameter independence, parameter modularity, and likelihood equivalence [24, 25].

#### 3.3.3. Structure Scoring and Search

To determine the Bayesian network or graphical model that can best fit the data, we needed a scoring system to compare different potential network structures. For structure learning, a Bayesian factor-based method, which compares the conditional probability of each graphical structure given observed data, was used. As shown below, according to Bayes theorem, the odds ratio between two possible structures, *G*_1_ and *G*_2_, can be decomposed as a product of structure prior odds ratio and the Bayesian factor, which is the ratio of the likelihoods of the two graphical models:

(2) $$\begin{array}{c} \frac{p\left(G_{1}\;|\;D\right)}{p\left(G_{2}\;|\;D\right)}=\frac{p\left(G_{1}\right)}{p\left(G_{2}\right)}*\frac{p\left(D\;|\;G_{1}\right)}{p\left(D\;|\;G_{2}\right)}. \end{array}$$

Using the uniform distribution for structure prior, which is reasonable because we have no preference on particular graphical structure, the score for a network structure, *G*, can be defined as the following formula, which is the log-likelihood of the graphical model:

(3) $$\begin{array}{c} \text{score}\left(G:D\right)=\log p\left(D\;|\;G\right)=\int p\left(D\;|\;\theta ,G\right)p\left(\theta \;|\;G\right)d\theta . \end{array}$$

In our study with 13 variables, there were 1.86766*e* + 31 possible directed acyclic graphs [26], so it was not realistic to enumerate the entire network structure space. To search more efficiently, we used a classical heuristic algorithm: hill climbing with random restarts [27, 28]. Using this stochastic algorithm, the search-space was reduced dramatically. Using 2 restarts, we only needed to compare 12, 655 structures before reaching a maximum score. One risk was that we had found a local maximum, rather than the global maximum, but the risk would be decreased further by increasing the number of restarts.

#### 3.3.4. Bootstrapping and Model Averaging

With the methods outlined above, we obtained a Bayesian network structure that best described the observed data. However, it is possible that the model may be overfitted, which means that a small change to the dataset could make the network structure change dramatically. A way to solve this issue is to apply a resampling method or simulating the dataset. The method would learn the best graphical model for each sampled dataset and generate a consensus network from the average of the sample models. This method is also known as model averaging. The simulation method we used to do model averaging was Efron's bootstrapping method [29, 30]. To increase robustness, the method only considered predicted network structures with a score within 95% of the confidence interval. The distribution of network scores is shown in Figure 5. In generating the final combined consensus network, edges were selected based on a confidence threshold of 75%.

### 3.4. Inferred Apoptosis Subnetwork

Using the described Gaussian Bayesian network modeling framework, a network model was generated for the 13 identified drug-responsive apoptotic genes as shown in Figure 6(a). The network contains 15 interactions and each edge has a confidence of over 75%. The inferred interactions represent dependence among these 13 genes of interest, which may be due to direct or indirect protein-protein interactions, transcriptional regulation, or signal transduction. To validate the inferred interactions, we searched the interactions component of NCBI Gene database (http://www.ncbi.nlm.nih.gov/gene), which contains data from multiple interaction databases such as BIND, HPRD, and BioGRID. We then generated a validated interaction network of the 13 apoptotic genes using their validated interactions (Figure 7). The validated network contained 216 interacting genes, including our 13 genes of interest. The network also contained 243 interactions after removing duplicate interactions (365 interactions with duplicates, Table S3). When compared with our predicted network, 9 out of 15 predicted interactions were found to be direct or indirect interactions in the validated network (marked in red, Figure 6(a)). An indirect interaction means the network does not contain a direct edge between the two genes, but there exists a path between them via intermediate genes.

Since we only considered 13 apoptotic genes in network inference, it is highly possible that the inferred interactions are indirect, but they illustrate the dependence or information transmission between the two corresponding genes. More precisely, a subvalidated network that includes only evidence (20 nodes and 28 interactions) for our predicted interactions was extracted as shown in Figure 6(b). For indirect evidence, we only counted the shortest paths between two apoptotic genes of interest.

#### 3.4.1. Known Direct Interactions

Two edges in our predicted network (marked in thick red, Figure 6(a)) have been validated as direct interactions in literature and are clearly annotated in the functional summary of corresponding genes as shown below.

*TAX1BP1 *→* TNFAIP3*. As seen in the annotation of TAX1BP1, Tax1 (human T-cell leukemia virus type I) binding protein 1, from the NCBI Gene database, this protein inhibits TNF-induced apoptosis by mediating TNFAIP3's antiapoptotic activity [31, 32].* In vivo* experiments and in vitro yeast two hybrid assays also confirm the interaction between TNFAIP3 (zinc finger protein A20) and TAX1BP1. TNFAIP3 also interacts with TXBP151, an antiapoptotic protein, and may inhibit inflammatory signaling pathways such as TNF-induced NF-*κ*B activation [33, 34]. TNFAIP3 and TAX1BP1 inhibit the inflammatory signaling pathway by interacting with Ubc13 and UbcH5c and triggering their ubiquitination and proteasome-dependent degradation [35].

*PMAIP1 *→* BCL2L11.* Although there is no evidence showing direct interaction between PMAIP1 (also known as NOXA) and BCL2L11 (also known as BIM), the functional annotation of PMAIP1 [36] from the UniProtKB/Swiss-Prot database shows that the PMAIP1 competes with BCL2L11 to bind with MCL1 and can displace BCL2L11 from its binding site on MCL1. The predicted interaction between PMAIP1 and BCL2L11 may be explained by the competition between PMAIP1 and BCL2L11 in binding MCL1. The competition may occur either through a direct interaction between the two proteins or through a third protein that is able to bind both. In addition, both PMAIP1 and BCL2L11 have been shown to interact directly with many other BCL2 protein family members including BCL2, BCL2A1, BCL2L1, and BCL2L2 [37, 38]. This indicates that NXOA and BIM may share common binding regions to BH3-only BCL2 family proteins. NOXA and BIM as BH3-only proteins have been recognized as critical mediators of anticancer drug- and p53-induced apoptotic responses [39, 40], which are consistent with our findings in this study that both of them are differentially expressed drug-responsive genes.

#### 3.4.2. Consistency with Two Major Cell Death Pathways

As described previously, there are two major apoptosis programs in mammalian cells: the intrinsic or mitochondrial stress-induced pathway and extrinsic or death receptor-triggered pathway. Our predicted network captures the important players and key interactions in both apoptosis programs. For the intrinsic pathway, our predicted network identifies two of the most important mediators, BLC2L11/BIM and PMAIP1/NOXA, and their competing interaction in terms of regulating many other BH3-only BCL2 family member proteins including BLC2, BCL2L1, BCL2L2, BCL2A1, and MCL1, which is illustrated as well in the validated network (Figure 6(b)). For the extrinsic death receptors-triggered pathway, we successfully recovered one representative of cancer-therapy or drug-induced cell death pathway: TNF-induced apoptosis. TNFAIP3/A20 and TAX1BP1/TXBP151 are two key players of this pathway, and they interact with each other to turn on the downstream cell death machinery.

#### 3.4.3. BCL2L11/BIM as a Gateway Gene to Drug-Induced Intrinsic Apoptosis

As shown in our inferred drug-induced apoptotic subnetwork, BCL2L11 is located downstream of most cell death subpathways, which includes drug-affected apoptotic genes such as BNIP3L, NOL3, PMAIP1, NUP62, and SON. This suggests that BCL2L11 may act as a downstream gate or switch for drug- or stress-induced apoptosis programs. This finding is consistent with the main role of BCL2L11 as an apoptosis facilitator. The mechanism through which BCL2L11, a BH3-only protein, activates cell death is by inactivating Bcl-2-like proteins, keeping them from restraining Bax and Bak. Bax or Bak can cause the outer membrane of the mitochondria to become permeable. This releases cytochrome c, which provokes Apaf-1 (apoptotic protease-activating factor 1) to activate caspase-9 [12]. The gateway role of BCL2L11 has also been illustrated in our literature-generated validation network (Figure 6(b)).

#### 3.4.4. TNFAIP3/A20 as a Gateway Gene to Drug-Induced Extrinsic Apoptosis

As shown in both our predicted network and validated network (Figure 6), TNFAIP3, a zinc finger protein, acts as a hub by transmitting upstream signals from cell death receptors to downstream cell death cascades. This suggests that TNFAIP3 may be a gateway protein for drug-induced extrinsic apoptosis. TNFAIP3/A20 acts as a key player in TNF-induced apoptosis by inhibiting NF-*κ*B activation. These results indicate that TNF-induced signaling may be the most common anticancer drug or chemical compound-triggered cell death program. Many studies have demonstrated the involvement of the TNF-mediated apoptosis in cancer therapies such as ionizing radiation or the chemotherapeutic agent, daunorubicin [31]. This again confirms that anticancer drugs induce apoptosis of cancer cells and that apoptosis pathways can be inferred from drug-perturbed gene expression profiles.

## 4. Discussion

We have demonstrated the value of CMAP data for studying drug-response in mammalian cancer cells. We have also validated the hypothesis that the apoptosis pathway may be a main program targeted by anticancer drugs. Furthermore, we have shown that CMAP data contains sufficient information about the dynamic activities of human genes for reconstructing gene-gene interactions in drug-perturbed cancer cells. We have also successfully applied a Gaussian Bayesian network framework to reconstruct a subnetwork containing validated interactions between genes with known roles in the apoptosis pathway. In addition, our network successfully predicted key players and interactions in drug-induced apoptosis, including both intrinsic and extrinsic apoptosis pathways.

**Figure 3:**
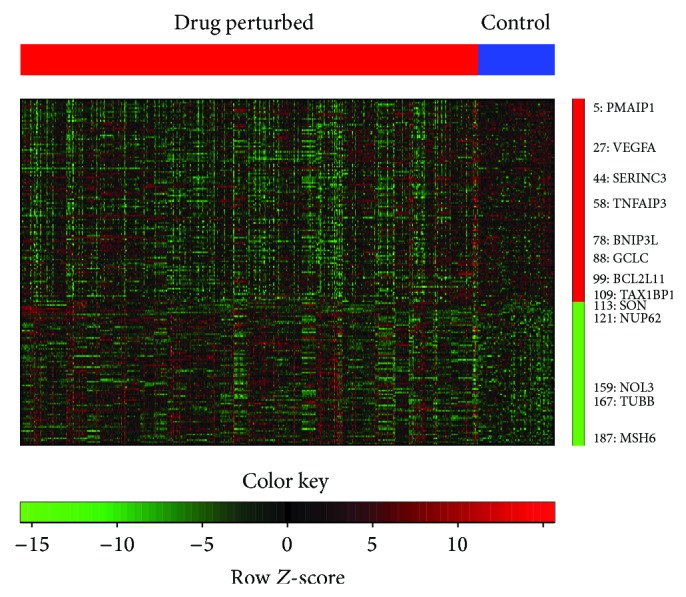
Heat map of top differentially expressed genes (FDR < 0.05) in drug-perturbed and control samples. The genes are ranked from most upregulated (labeled in dark red on right panel) to most downregulated (labeled in dark green) in drug-perturbed samples, and the 13 selected apoptotic genes are labeled on the right with their ranks in the list.

**Figure 4:**
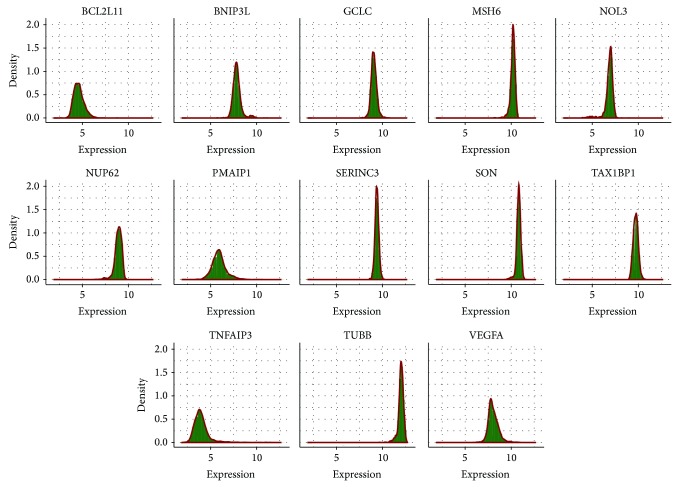
Marginal distributions of the 13 selected drug-responsive apoptotic genes across all samples in CMAP data.

**Figure 5:**
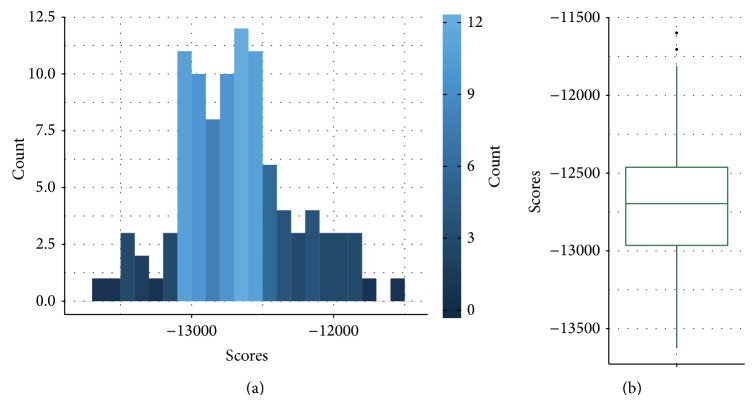
(a) Histogram and (b) box plot of scores for best-learned graphical model in each bootstrapped sampling.

**Figure 6:**
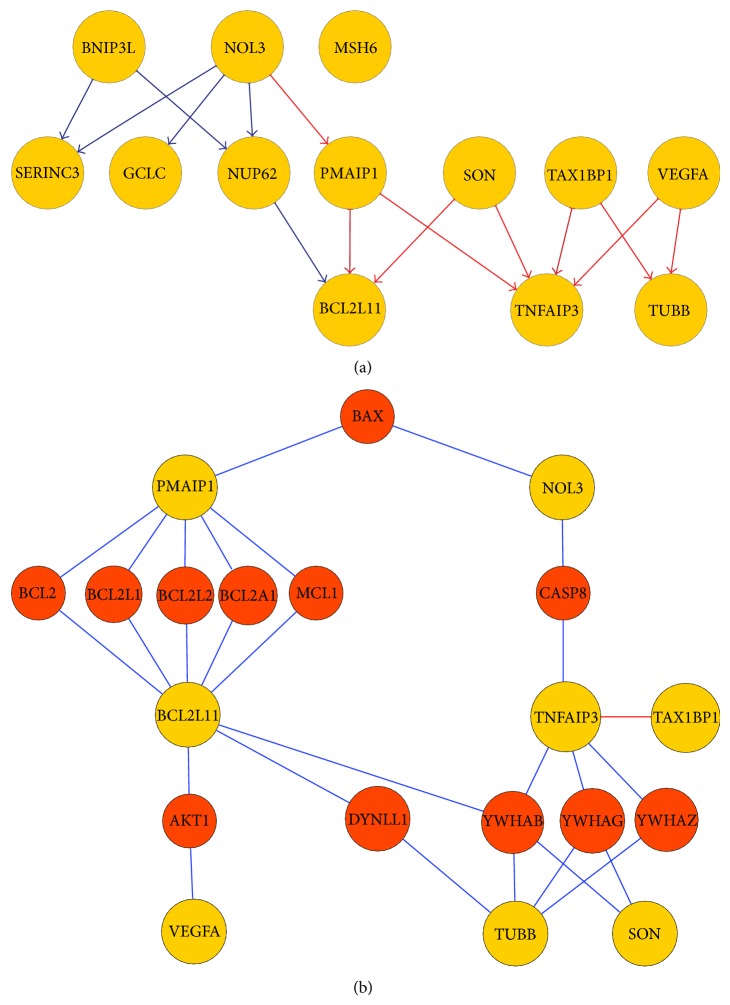
(a) Predicted subnetwork of 13 selected drug-responsive apoptotic genes: edges in red are validated interactions in literature and edges in dark red are strong validated direct interactions. (b) A subnetwork from literature showing evidences for validated interactions in predicted network including candidate genes (colored in yellow) with their validated interactants (in brown). Each validated edge in predicted network (red in (a)) can be mapped to one path in evidence network (b) between the two corresponding interacting candidate genes.

**Figure 7:**
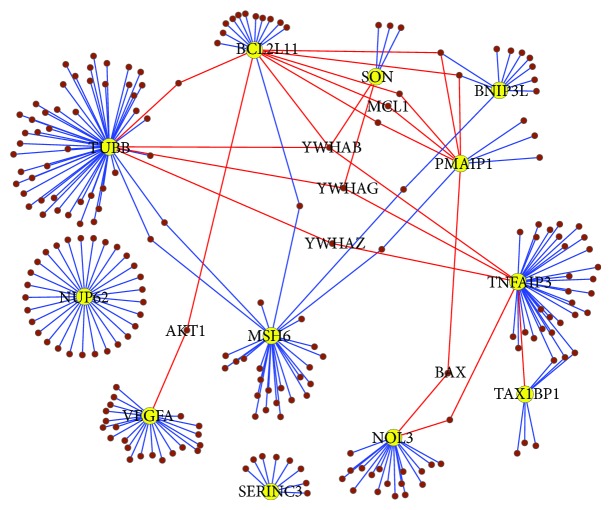
A network from literature for 13 candidate genes (colored in yellow) with their validated interaction candidates (in brown). Edges in red are evidence for validation of interactions in predicted apoptosis network.

Our framework may be improved in a few ways. First, we only considered the general effects of drugs based on the assumption that cancer cells have a similar response mechanism to different drugs. However this assumption may be overgeneralized, since there are some drugs to which the cells have no response. This can be clearly seen in Figure 3, which contains a heat map of signature genes across all drugs. One way to deal with this limitation may be to cluster drugs by their expression profiles or by their physical or chemical properties. A similar comparison analysis may be performed but would take into account the effects of different drug groups. Second, to reduce computational complexity, we limited our analysis to apoptotic genes that were differentially expressed with a Bonferroni-corrected *P* value threshold of 0.05. This threshold might have been overly stringent and may have caused us to filter informative genes from the analysis. One way to deal with this problem might be to include more candidate genes, but this would increase complexity and computation.

We have shown that Bayesian network modeling can be a powerful tool for reconstructing biological networks from noisy high-throughput microarray data. In the Bayesian network modeling approach to network reconstruction, we have found that a linear Gaussian model for local probability distribution is able to give a more accurate description for continuous data and is also able to reduce the number of parameters when compared to discrete methods. In discrete methods, data points are separated into multiple levels, and this can result in the loss of information, especially in cases where the variable has a large range of values and has many parent variables [10–14]. However, one limitation with the linear Gaussian model is that although it works well in cases where the data fits a normal distribution and there are linear dependencies between nodes and their parents, the model can easily overfit the data if these dependencies are not met. In this study it was reasonable to apply Gaussian distribution because most candidate genes fit a normal distribution, as shown in Figure 1. However, a possible improvement may result from performing graphical diagnosis and further transformation on the data or employing other statistical models to fit the data. An alternative approach to deconvoluting the structure of the Bayesian network is simulated annealing with Markov chain Monte Carlo (MCMC) sampling. This method may overcome the limitation of the hill-climbing method used in this study. In hill-climbing method, the function finds the nearest optimum value. Depending on the starting point, this peak may or may not be the true optimum value. However, one limitation with MCMC sampling is that it is significantly more time-consuming than the hill-climbing method. For network comparison or scoring, other asymptotic criteria such as AIC, BIC, or DIC could be tried as well.

The two major apoptosis subpathways of mammalian cells are largely independent because overexpressed Bcl-2 does not protect lymphocytes from apoptosis induced by death receptor ligands. Literature has shown that in certain other cell types, such as hepatocytes, the two pathways intersect; CASP8 can process the proapoptotic Bid into its active truncated form (tBid) and prevent catastrophic untimely cell death [11]. However, cross talk between these two programs has been rarely studied in the context of drug-perturbations. Our computationally predicted apoptosis network might shed light on how both pathways are regulated together by identifying cross talk interactions such as PMAIP1 and TNFAIP3 and BCL2L11 and TNFAIP3 via SON.

In summary, we have extended the usage of CMAP data and reconstructed a subnetwork of drug-induced apoptosis in mammalian cancer cells using a computational statistical modeling approach. Our findings have added new knowledge of how cancer cells respond to compounds and provide potential specific targets in the apoptosis pathway for tailored therapeutics. Additionally, as a final consideration, cell death might not be the only drug-induced program in cell response, so our computational framework to CMAP data could be extended to other interesting biological pathways related to cancer treatment by drugs.

## 5. Conclusions

Anticancer therapeutic drugs are designed to induce tumor-selective cell death programs. Hence, it is critical to understand what specific apoptosis proteins and pathways are stimulated in cancer cells by chemical compounds and how these pathways act. The Connectivity Map (CMAP) project, aiming to connect diseases, genes, and drugs, makes it possible to address this question systematically, with large-scaled genome-wide gene expression profiles of human tumor cells exposed to a library of anticancer compounds. Using the CMAP data, we confirm that indeed cell death is one major program trigged by anticancer agents. Furthermore, we demonstrate that the drug-induced cell death subnetwork can be computationally inferred using a Bayesian network modeling approach. Our predicted subnetwork successfully captures the two major intrinsic and extrinsic cell death pathways and identifies key “gateway” players and interactions in each. This study provides a computational framework to recover underlying drug-induced biological networks from* perturbation of gene expression data* and to better understand the mechanism of action driving drug compound effects on cells.

## Supplementary Material

In the supplementary files, we provide details about the top differentially-expressed genes induced by anti-cancer drugs (Table S1), and their enrichment in pro- or anti- apoptotic gene sets (Figure S1-2, Table S2). We also provide details of literature-identified interactions among the 13 selected candidate apoptotic genes (Table S3).

## Figures and Tables

**Table 1:** The 13 selected differentially expressed or drug-responsive apoptotic genes.

Gene symbol	probeId	entrezId	log⁡FC	*t*	*P* value	FDR	Apoptosis type^*^
PMAIP1	204285_s_at	5366	0.32	7.46	1.19*E* − 13	2.64*E* − 09	pro
VEGFA	210512_s_at	7422	0.18	6.15	8.88*E* − 10	1.98*E* − 05	anti
SERINC3	221471_at	10955	0.06	5.62	2.08*E* − 08	4.64*E* − 04	pro
TNFAIP3	202644_s_at	7128	0.24	5.39	7.61*E* − 08	1.70*E* − 03	anti
BNIP3L	221479_s_at	665	0.12	5.04	4.88*E* − 07	1.09*E* − 02	both
GCLC	202923_s_at	2729	0.08	4.91	9.42*E* − 07	2.10*E* − 02	anti
BCL2L11	222343_at	10018	0.14	4.83	1.41*E* − 06	3.14*E* − 02	pro
TAX1BP1	200976_s_at	8887	0.07	4.76	2.01*E* − 06	4.47*E* − 02	anti
SON	214988_s_at	6651	−0.06	−4.75	2.11*E* − 06	4.71*E* − 02	anti
NUP62	202153_s_at	23636	−0.11	−4.83	1.41*E* − 06	3.14*E* − 02	anti
NOL3	59625_at	8996	−0.13	−5.32	1.13*E* − 07	2.53*E* − 03	anti
TUBB	212320_at	203068	−0.09	−5.55	3.11*E* − 08	6.92*E* − 04	pro
MSH6	202911_at	2956	−0.09	−6.40	1.87*E* − 10	4.16*E* − 06	pro

^*^pro: annotated by GO terms: induction of apoptosis, positive regulation of apoptosis, and negative regulation of antiapoptosis; anti: annotated by GO terms: negative regulation of apoptosis and positive regulation of antiapoptosis.
