# Supplementary material for: Recovering Drug-Induced Apoptosis Subnetwork from Connectivity Map Data
Source: Biomed Res Int. 2015 Mar 25;2015:708563. doi: 10.1155/2015/708563 (PMC4389823; doi:10.1155/2015/708563)
Supplement: Supplementary file 1 — In the supplementary files, we provide details about the top differentially-expressed genes induced by anti-cancer drugs (Table S1), and their enrichment in pro- or anti- apoptotic gene sets (Figure S1-2, Table S2). We also provide details of literature-identified interactions among the 13 selected candidate apoptotic genes (Table S3). [file 708563.f1.pdf]

Figure Supp.1

A

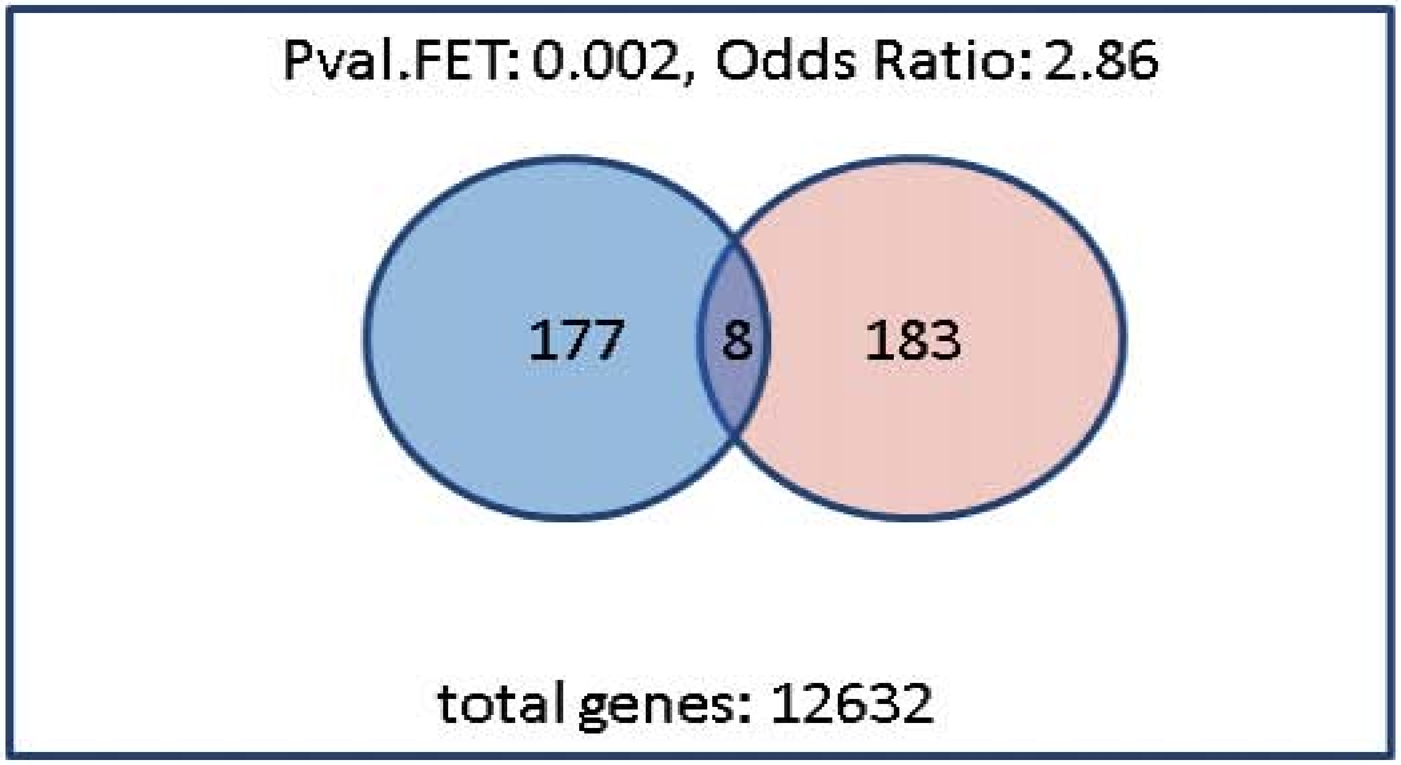

B

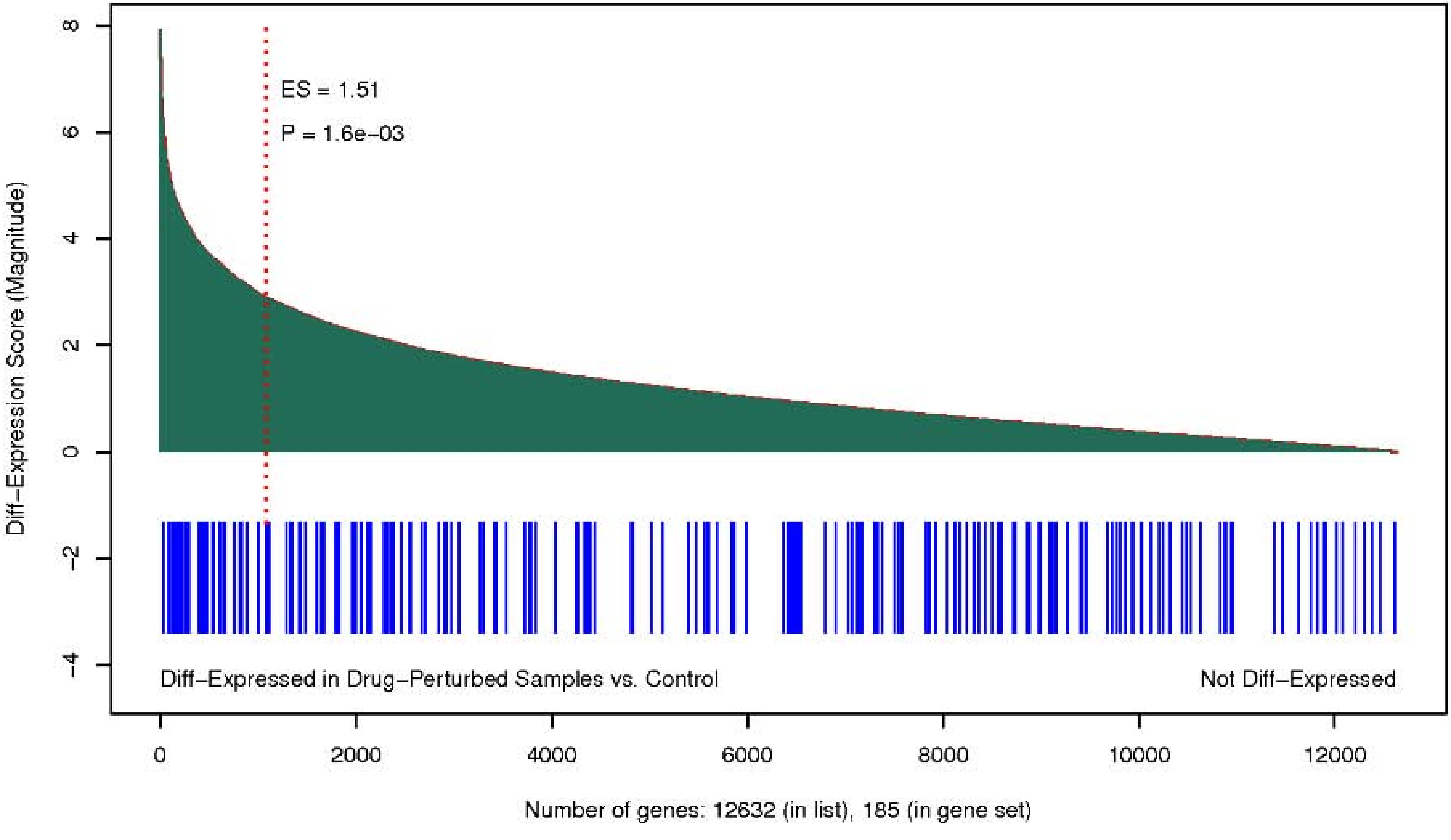

Figure Supp.2

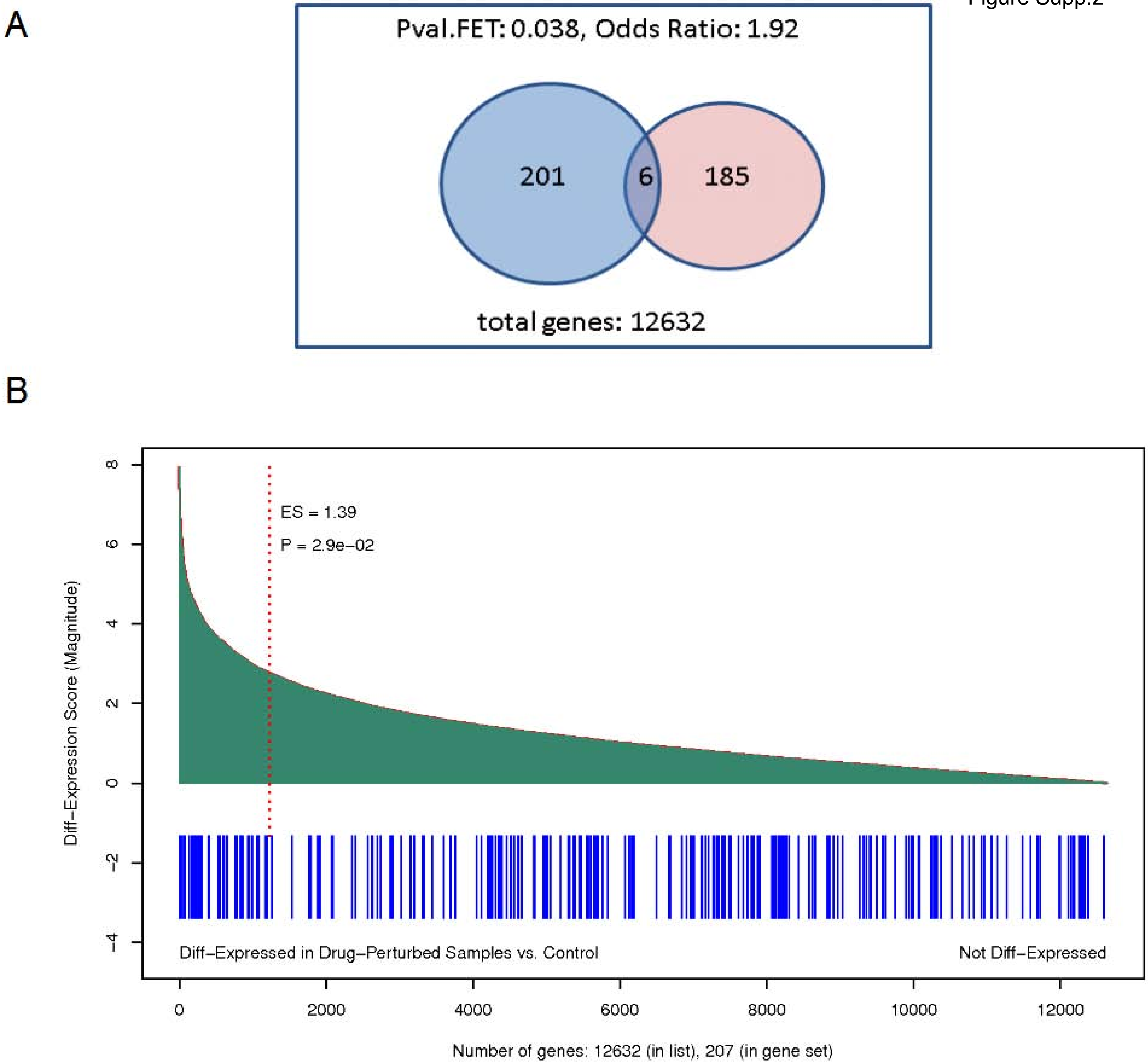

Supplementary Table S1: The 191 differentially-expressed drug-responsive si

probeId	geneSymbol	entrezId	logFC	aveExpr	t	P.Value
36711_at	MAFF	23764	0.55	4.09	8.05	1.18E-15
202672_s_at	ATF3	467	0.54	3.87	7.93	3.17E-15
202887_s_at	DDIT4	54541	0.30	8.77	7.53	6.83E-14
203455_s_at	SAT1	6303	0.25	7.28	7.51	7.78E-14
204285_s_at	PMAIP1	5366	0.32	5.93	7.46	1.19E-13
217168_s_at	HERPUD1	9709	0.18	8.91	7.34	2.86E-13
208786_s_at	MAP1LC3B	81631	0.19	9.39	7.25	5.47E-13
202770_s_at	CCNG2	901	0.30	5.36	7.22	6.78E-13
205047_s_at	ASNS	440	0.14	10.12	7.10	1.55E-12
219270_at	CHAC1	79094	0.27	3.43	6.93	5.13E-12
201694_s_at	EGR1	1958	0.54	3.80	6.85	8.88E-12
202557_at	HSPA13	6782	0.19	7.83	6.79	1.32E-11
209383_at	DDIT3	1649	0.29	4.65	6.76	1.72E-11
206277_at	P2RY2	5029	-0.20	5.65	-6.73	1.98E-11
209121_x_at	NR2F2	7026	-0.19	9.57	-6.70	2.52E-11
203725_at	GADD45A	1647	0.21	7.82	6.69	2.67E-11
214141_x_at	SFRS7	6432	-0.11	11.67	-6.62	4.22E-11
201266_at	TXNRD1	7296	0.11	10.80	6.46	1.22E-10
212623_at	TMEM41B	440026	0.13	8.86	6.46	1.22E-10
212888_at	DICER1	23405	-0.10	9.31	-6.43	1.46E-10
217678_at	SLC7A11	23657	0.30	6.28	6.41	1.71E-10
209146_at	SC4MOL	6307	0.13	10.76	6.40	1.81E-10
202911_at	MSH6	2956	-0.09	10.17	-6.40	1.87E-10
209185_s_at	IRS2	8660	0.21	3.47	6.39	1.92E-10
216248_s_at	NR4A2	4929	0.18	3.27	6.38	2.03E-10
221985_at	KLHL24	54800	0.30	4.34	6.38	2.05E-10
209102_s_at	HBP1	26959	0.16	7.38	6.38	2.09E-10
215945_s_at	TRIM2	23321	0.15	3.69	6.36	2.39E-10
212274_at	LPIN1	23175	0.24	8.22	6.33	2.90E-10
202727_s_at	IFNGR1	3459	0.13	8.38	6.31	3.15E-10
202613_at	CTPS	1503	-0.12	9.00	-6.30	3.43E-10
219127_at	ATAD4	79170	-0.26	7.27	-6.27	4.07E-10
219681_s_at	RAB11FIP1	80223	0.14	7.24	6.19	6.90E-10
210512_s_at	VEGFA	7422	0.18	8.05	6.15	8.88E-10
202679_at	NPC1	4864	0.17	8.17	6.13	1.00E-09
201627_s_at	INSIG1	3638	0.20	8.57	6.12	1.05E-09
220081_x_at	HSD17B7	51478	0.13	7.78	6.07	1.44E-09
202722_s_at	GFPT1	2673	0.10	9.03	6.05	1.59E-09
208881_x_at	IDI1	3422	0.12	11.22	6.05	1.62E-09
202211_at	ARFGAP3	26286	0.16	7.45	6.05	1.68E-09
202539_s_at	HMGCR	3156	0.16	8.42	6.04	1.76E-09
219798_s_at	MEPCE	56257	-0.18	9.42	-6.03	1.85E-09
218858_at	DEPDC6	64798	0.19	4.89	6.01	2.09E-09
208804_s_at	SFRS6	6431	-0.09	9.47	-5.98	2.56E-09
212798_s_at	ANKMY2	57037	0.14	7.96	5.97	2.60E-09
205527_s_at	GEMIN4	50628	-0.16	8.16	-5.96	2.91E-09

217562_at	FAM5C	339479	-0.31	6.55	-5.88	4.45E-09
37028_at	PPP1R15A	23645	0.19	3.69	5.86	5.19E-09
209124_at	MYD88	4615	-0.12	8.78	-5.86	5.24E-09
204695_at	CDC25A	993	-0.13	5.99	-5.82	6.54E-09
204472_at	GEM	2669	0.26	2.81	5.82	6.69E-09
212124_at	ZMIZ1	57178	-0.12	10.21	-5.73	1.09E-08
221577_x_at	GDF15	9518	0.35	8.34	5.73	1.13E-08
209238_at	STX3	6809	0.10	8.88	5.72	1.19E-08
215165_x_at	UMPS	7372	-0.10	8.86	-5.71	1.22E-08
202074_s_at	OPTN	10133	0.22	5.28	5.64	1.84E-08
218653_at	SLC25A15	10166	-0.11	8.83	-5.64	1.84E-08
205356_at	USP13	8975	-0.10	8.79	-5.63	1.93E-08
214657_s_at	NCRNA00084	283131	0.25	3.30	5.63	2.03E-08
203721_s_at	UTP18	51096	-0.08	10.95	-5.62	2.04E-08
212233_at	MAP1B	4131	0.21	3.19	5.62	2.04E-08
221471_at	SERINC3	10955	0.06	9.39	5.62	2.08E-08
209494_s_at	PATZ1	23598	-0.19	5.85	-5.61	2.22E-08
201925_s_at	CD55	1604	0.18	8.04	5.61	2.23E-08
218611_at	IER5	51278	0.14	9.62	5.60	2.31E-08
41037_at	TEAD4	7004	-0.14	5.68	-5.60	2.41E-08
201761_at	MTHFD2	10797	0.07	11.76	5.58	2.59E-08
201586_s_at	SFPQ	6421	-0.06	11.24	-5.57	2.71E-08
205413_at	MPPED2	744	-0.22	7.79	-5.57	2.76E-08
208933_s_at	LGALS8	3964	0.12	6.55	5.57	2.82E-08
211784_s_at	SFRS1	6426	-0.06	12.35	-5.57	2.85E-08
202107_s_at	MCM2	4171	-0.07	10.65	-5.56	3.03E-08
221750_at	HMGCS1	3157	0.14	8.54	5.55	3.04E-08
217858_s_at	ARMCX3	51566	0.09	9.63	5.55	3.07E-08
212320_at	TUBB	203068	-0.09	12.00	-5.55	3.11E-08
212335_at	GNS	2799	0.10	8.66	5.49	4.30E-08
204326_x_at	MT1X	4501	0.21	8.91	5.49	4.49E-08
210999_s_at	GRB10	2887	0.15	4.00	5.48	4.74E-08
203666_at	CXCL12	6387	-0.30	6.48	-5.47	4.81E-08
205241_at	SCO2	9997	-0.16	7.88	-5.46	5.11E-08
203640_at	MBNL2	10150	0.17	6.69	5.46	5.22E-08
203811_s_at	DNAJB4	11080	0.23	4.06	5.43	6.22E-08
211423_s_at	SC5DL	6309	0.09	9.47	5.43	6.25E-08
221613_s_at	ZFAND6	54469	0.06	10.06	5.42	6.29E-08
202809_s_at	INTS3	65123	-0.12	7.47	-5.40	7.29E-08
221235_s_at	LOC644617	644617	-0.13	6.15	-5.39	7.42E-08
202644_s_at	TNFAIP3	7128	0.24	3.97	5.39	7.61E-08
212689_s_at	JMJD1A	55818	0.14	7.06	5.37	8.44E-08
212330_at	TFDP1	7027	-0.07	9.90	-5.33	1.07E-07
213762_x_at	RBMX	27316	-0.06	11.24	-5.32	1.10E-07
218769_s_at	ANKRA2	57763	0.14	6.34	5.32	1.11E-07
212022_s_at	MKI67	4288	-0.09	9.89	-5.32	1.13E-07
59625_at	NOL3	8996	-0.13	6.84	-5.32	1.13E-07
218300_at	C16orf53	79447	-0.13	8.71	-5.32	1.15E-07
218993_at	RNMTL1	55178	-0.08	8.94	-5.29	1.30E-07
221069_s_at	CCDC44	51204	-0.08	9.61	-5.29	1.35E-07

208961_s_at	KLF6	1316	0.19	6.18	5.26	1.55E-07
205660_at	OASL	8638	0.21	2.57	5.25	1.59E-07
219186_at	ZBTB7A	51341	-0.10	4.33	-5.25	1.65E-07
218976_at	DNAJC12	56521	0.21	6.85	5.25	1.65E-07
202364_at	MXI1	4601	0.15	7.14	5.23	1.82E-07
209267_s_at	SLC39A8	64116	-0.08	10.39	-5.22	1.96E-07
205258_at	INHBB	3625	-0.18	9.72	-5.20	2.11E-07
218364_at	LRRFIP2	9209	0.10	6.23	5.20	2.11E-07
220192_x_at	SPDEF	25803	-0.14	10.70	-5.20	2.13E-07
213237_at	C16orf88	400506	-0.10	7.44	-5.20	2.16E-07
218618_s_at	FNDC3B	64778	0.12	8.19	5.20	2.18E-07
214635_at	CLDN9	9080	-0.19	4.91	-5.19	2.23E-07
202333_s_at	UBE2B	7320	0.07	9.98	5.19	2.29E-07
212993_at	NACC2	138151	-0.14	8.05	-5.18	2.35E-07
210766_s_at	CSE1L	1434	-0.05	11.08	-5.18	2.37E-07
213417_at	TBX2	6909	-0.16	8.55	-5.18	2.38E-07
200768_s_at	MAT2A	4144	-0.11	9.80	-5.18	2.39E-07
209189_at	FOS	2353	0.21	2.44	5.18	2.42E-07
210239_at	IRX5	10265	-0.18	8.35	-5.14	2.87E-07
202842_s_at	DNAJB9	4189	0.16	8.40	5.14	2.88E-07
200875_s_at	NOP56	10528	-0.08	10.36	-5.14	2.94E-07
203625_x_at	SKP2	6502	-0.11	9.06	-5.13	3.05E-07
203217_s_at	ST3GAL5	8869	0.19	3.64	5.13	3.16E-07
203821_at	HBEGF	1839	0.15	3.03	5.10	3.64E-07
221962_s_at	UBE2H	7328	0.15	6.48	5.09	3.88E-07
205061_s_at	EXOSC9	5393	-0.09	8.60	-5.08	4.02E-07
212470_at	SPAG9	9043	0.12	8.39	5.08	4.06E-07
213397_x_at	RNASE4	6038	0.13	2.05	5.07	4.26E-07
221291_at	ULBP2	80328	0.21	3.35	5.07	4.27E-07
208056_s_at	CBFA2T3	863	-0.22	8.79	-5.06	4.45E-07
212907_at	SLC30A1	7779	0.18	7.79	5.05	4.63E-07
213682_at	NUP50	10762	-0.07	9.21	-5.05	4.72E-07
204488_at	DOLK	22845	-0.12	7.71	-5.05	4.78E-07
202720_at	TES	26136	0.15	7.97	5.05	4.80E-07
221479_s_at	BNIP3L	665	0.12	7.86	5.04	4.88E-07
200673_at	LAPTM4A	9741	0.05	11.45	5.04	4.99E-07
204352_at	TRAF5	7188	-0.14	4.91	-5.02	5.44E-07
202126_at	PRPF4B	8899	-0.08	9.43	-5.02	5.63E-07
202710_at	BET1	10282	0.09	8.30	5.00	6.09E-07
205870_at	BDKRB2	624	0.10	2.56	5.00	6.18E-07
218853_s_at	MOSPD1	56180	0.09	8.98	4.97	7.08E-07
202388_at	RGS2	5997	0.25	3.22	4.97	7.10E-07
202429_s_at	PPP3CA	5530	-0.05	11.04	-4.95	7.68E-07
218982_s_at	MRPS17	51373	-0.06	9.73	-4.95	7.87E-07
207543_s_at	P4HA1	5033	0.09	7.75	4.95	7.93E-07
208974_x_at	KPNB1	3837	-0.07	11.53	-4.94	8.20E-07
212698_s_at	10-Sep	151011	0.20	4.57	4.93	8.63E-07
219650_at	ERCC6L	54821	-0.12	7.20	-4.93	8.72E-07
204441_s_at	POLA2	23649	-0.09	7.81	-4.92	8.96E-07
201377_at	UBAP2L	9898	-0.07	10.09	-4.92	8.96E-07

203665_at	HMOX1	3162	0.32	3.78	4.92	9.02E-07
203232_s_at	ATXN1	6310	0.12	7.12	4.92	9.08E-07
46665_at	SEMA4C	54910	-0.18	9.80	-4.91	9.39E-07
202923_s_at	GCLC	2729	0.08	9.07	4.91	9.42E-07
219258_at	TIPIN	54962	-0.09	9.29	-4.91	9.56E-07
219084_at	NSD1	64324	-0.11	5.33	-4.91	9.60E-07
218319_at	PELI1	57162	0.13	4.58	4.90	9.97E-07
218581_at	ABHD4	63874	0.13	3.88	4.90	1.00E-06
219990_at	E2F8	79733	-0.12	8.00	-4.89	1.07E-06
204059_s_at	ME1	4199	0.06	11.01	4.89	1.08E-06
209336_at	PWP2	5822	-0.10	7.61	-4.89	1.08E-06
217832_at	SYNCRIP	10492	-0.07	10.77	-4.89	1.08E-06
209479_at	CCDC28A	25901	0.14	6.86	4.88	1.10E-06
201041_s_at	DUSP1	1843	0.19	6.28	4.88	1.11E-06
219968_at	ZNF589	51385	-0.07	3.13	-4.88	1.12E-06
212239_at	PIK3R1	5295	-0.16	8.88	-4.88	1.13E-06
221658_s_at	IL21R	50615	0.09	2.27	4.88	1.15E-06
200631_s_at	SET	6418	-0.04	11.91	-4.87	1.17E-06
201170_s_at	BHLHE40	8553	0.21	6.08	4.87	1.19E-06
205807_s_at	TUFT1	7286	0.18	8.69	4.87	1.20E-06
217783_s_at	YPEL5	51646	0.15	7.79	4.86	1.23E-06
203827_at	WIPI1	55062	0.19	5.61	4.84	1.39E-06
202153_s_at	NUP62	23636	-0.11	8.87	-4.83	1.41E-06
222343_at	BCL2L11	10018	0.14	4.60	4.83	1.41E-06
217165_x_at	MT1F	4494	0.20	5.81	4.83	1.42E-06
218103_at	FTSJ3	117246	-0.06	9.87	-4.83	1.42E-06
204510_at	CDC7	8317	-0.12	6.82	-4.83	1.45E-06
219356_s_at	CHMP5	51510	0.06	10.63	4.81	1.55E-06
209345_s_at	PI4K2A	55361	0.15	5.47	4.81	1.59E-06
221489_s_at	SPRY4	81848	0.07	2.18	4.79	1.73E-06
209567_at	RRS1	23212	-0.13	10.14	-4.78	1.85E-06
33304_at	ISG20	3669	0.13	3.66	4.78	1.86E-06
221563_at	DUSP10	11221	0.15	3.64	4.78	1.87E-06
218865_at	MOSC1	64757	-0.11	6.88	-4.78	1.88E-06
212942_s_at	KIAA1199	57214	0.14	2.82	4.78	1.89E-06
221549_at	GRWD1	83743	-0.12	6.27	-4.77	1.93E-06
219581_at	TSEN2	80746	-0.11	7.31	-4.77	1.95E-06
204979_s_at	SH3BGR	6450	0.16	5.14	4.76	1.99E-06
214748_at	N4BP2L2	10443	0.16	3.53	4.76	1.99E-06
200976_s_at	TAX1BP1	8887	0.07	9.72	4.76	2.01E-06
218001_at	MRPS2	51116	-0.08	9.94	-4.76	2.06E-06
203153_at	IFIT1	3434	0.25	2.69	4.75	2.09E-06
214988_s_at	SON	6651	-0.06	10.81	-4.75	2.11E-06
209610_s_at	SLC1A4	6509	0.17	5.73	4.75	2.11E-06
201397_at	PHGDH	26227	0.10	7.58	4.75	2.16E-06

gnature genes (FDR<0.05)

FDR (<0.05)
2.63E-11
7.07E-11
1.52E-09
1.73E-09
2.64E-09
6.36E-09
1.22E-08
1.51E-08
3.46E-08
1.14E-07
1.98E-07
2.95E-07
3.84E-07
4.42E-07
5.61E-07
5.94E-07
9.40E-07
2.71E-06
2.72E-06
3.26E-06
3.80E-06
4.04E-06
4.16E-06
4.28E-06
4.52E-06
4.58E-06
4.65E-06
5.32E-06
6.45E-06
7.03E-06
7.63E-06
9.07E-06
1.54E-05
1.98E-05
2.24E-05
2.34E-05
3.20E-05
3.55E-05
3.61E-05
3.74E-05
3.93E-05
4.13E-05
4.65E-05
5.70E-05
5.80E-05
6.49E-05

9.92E-05
1.16E-04
1.17E-04
1.46E-04
1.49E-04
2.43E-04
2.52E-04
2.65E-04
2.72E-04
4.09E-04
4.10E-04
4.31E-04
4.52E-04
4.55E-04
4.55E-04
4.64E-04
4.94E-04
4.97E-04
5.14E-04
5.37E-04
5.77E-04
6.04E-04
6.14E-04
6.27E-04
6.35E-04
6.74E-04
6.77E-04
6.84E-04
6.92E-04
9.58E-04
1.00E-03
1.06E-03
1.07E-03
1.14E-03
1.16E-03
1.39E-03
1.39E-03
1.40E-03
1.62E-03
1.65E-03
1.70E-03
1.88E-03
2.38E-03
2.46E-03
2.48E-03
2.52E-03
2.53E-03
2.55E-03
2.91E-03
3.00E-03

3.44E-03
3.54E-03
3.67E-03
3.68E-03
4.06E-03
4.36E-03
4.70E-03
4.71E-03
4.74E-03
4.80E-03
4.85E-03
4.96E-03
5.11E-03
5.23E-03
5.29E-03
5.31E-03
5.32E-03
5.39E-03
6.39E-03
6.42E-03
6.54E-03
6.80E-03
7.04E-03
8.11E-03
8.65E-03
8.96E-03
9.04E-03
9.49E-03
9.52E-03
9.91E-03
1.03E-02
1.05E-02
1.07E-02
1.07E-02
1.09E-02
1.11E-02
1.21E-02
1.25E-02
1.36E-02
1.38E-02
1.58E-02
1.58E-02
1.71E-02
1.75E-02
1.77E-02
1.83E-02
1.92E-02
1.94E-02
2.00E-02
2.00E-02

2.01E-02
2.02E-02
2.09E-02
2.10E-02
2.13E-02
2.14E-02
2.22E-02
2.23E-02
2.38E-02
2.40E-02
2.41E-02
2.42E-02
2.46E-02
2.47E-02
2.49E-02
2.52E-02
2.56E-02
2.61E-02
2.66E-02
2.68E-02
2.74E-02
3.09E-02
3.14E-02
3.14E-02
3.17E-02
3.17E-02
3.23E-02
3.45E-02
3.55E-02
3.84E-02
4.12E-02
4.15E-02
4.17E-02
4.19E-02
4.21E-02
4.29E-02
4.35E-02
4.43E-02
4.44E-02
4.47E-02
4.58E-02
4.65E-02
4.71E-02
4.71E-02
4.81E-02

Supplementary Table S3: The 380 GO-annotated apoptotic genes, out of which 368 ge

geneSymbol	type	hasMicroarrayData
ABL1	pro	YES
ACVR1B	pro	YES
ADAMTSL4	pro	YES
ADORA1	pro	YES
AIFM1	pro	YES
ALOX15B	pro	YES
APBB2	both	YES
APOE	pro	YES
B4GALT1	pro	YES
BAD	pro	YES
BAK1	pro	YES
BARD1	both	YES
BAX	both	YES
BBC3	pro	YES
BCL10	both	YES
BCL2L11	pro	YES
BCL2L13	pro	YES
BCL3	both	YES
BCL6	both	YES
BCLAF1	pro	YES
BID	pro	YES
BIK	pro	YES
BNIP1	both	YES
BNIP3	both	YES
BNIP3L	both	YES
BOK	pro	YES
BRCA1	pro	YES
BTK	pro	YES
C16orf5	pro	YES
CADM1	pro	YES
CASP10	pro	YES
CASP3	both	YES
CASP4	pro	YES
CASP6	pro	YES
CASP8AP2	pro	YES
CD2	pro	YES
CD24	pro	YES
CD27	both	YES
CD28	both	YES
CD38	pro	YES
CD5	pro	YES
CD70	pro	YES
CDK5	pro	YES

CDK5R1	pro	YES
CDKN1A	both	YES
CEBPB	both	YES
CEBPG	pro	YES
CFLAR	both	YES
CHEK2	pro	YES
CIDEA	pro	YES
CIDEB	pro	YES
CIDEC	pro	YES
COL18A1	pro	YES
COL4A3	pro	YES
CRADD	pro	YES
CRTAM	pro	YES
CUL1	pro	YES
CUL2	pro	YES
CUL3	pro	YES
CUL4A	pro	YES
CUL5	pro	YES
DAP	pro	YES
DAP3	pro	YES
DAPK1	pro	YES
DAPK2	pro	YES
DAPK3	pro	YES
DAXX	pro	YES
DCC	pro	YES
DEDD	pro	YES
DIABLO	pro	YES
DNM2	pro	YES
DPF1	pro	YES
DPF2	pro	YES
DYRK2	pro	YES
EEF1E1	pro	YES
EI24	pro	YES
ERCC2	pro	YES
ERCC3	pro	YES
ERN1	pro	YES
ERN2	pro	YES
FADD	pro	YES
FAF1	pro	YES
FAS	both	YES
FASLG	pro	YES
FASTK	pro	YES
FEM1B	pro	YES
FOXL2	pro	YES
FOXO3	pro	YES
GPX1	pro	YES
HD	both	NO

HIPK2	pro	YES
HRK	both	YES
HTATIP2	both	YES
IFNA2	pro	YES
IFNB1	pro	YES
IGFBP3	pro	YES
IHPK2	pro	NO
IKBKG	pro	YES
IL18	pro	YES
IL19	pro	YES
INHA	pro	YES
INHBA	pro	YES
KNG1	pro	YES
LAG3	pro	YES
LALBA	pro	YES
LCK	pro	YES
LTA	pro	YES
LTB4R2	pro	YES
MAL	pro	YES
MAP3K10	pro	YES
MAP3K5	pro	YES
MAPK1	pro	YES
MBD4	pro	YES
MMP9	pro	YES
MSH6	pro	YES
MTCH1	pro	YES
MX1	pro	YES
MYC	both	YES
NCR1	pro	YES
NDUFA13	pro	YES
NDUFS3	pro	YES
NF1	pro	YES
NGFR	pro	YES
NLRP1	pro	YES
NLRP3	pro	YES
NME3	pro	YES
NOTCH2	both	YES
NOX5	pro	YES
NUDT2	pro	YES
NUPR1	pro	YES
PAWR	pro	YES
PCBP4	pro	YES
PDCD5	pro	YES
PDCD6	pro	YES
PDIA3	pro	YES
PLAGL1	pro	YES
PLAGL2	pro	YES

PLG	pro	YES
PMAIP1	pro	YES
PML	pro	YES
POU4F1	pro	NO
PPP1R13B	pro	YES
PPP2CA	pro	YES
PPP2R1A	pro	YES
PPP2R1B	pro	YES
PRKCA	pro	YES
PRKCE	pro	YES
PRKRA	pro	YES
PRODH	pro	YES
PSEN2	pro	YES
PTEN	both	YES
PTH	pro	YES
PTPRC	pro	YES
PYCARD	pro	YES
PYHIN1	pro	YES
RAB27A	pro	YES
RAD9A	pro	YES
RARG	pro	YES
RIPK2	pro	YES
RNF7	both	YES
RPS3A	pro	YES
RUNX3	pro	YES
S100B	pro	YES
SAP30BP	pro	YES
SART1	pro	YES
SCIN	pro	YES
SERINC3	pro	YES
SFN	pro	YES
SIPA1	pro	YES
SIVA1	pro	YES
SLAMF7	pro	YES
SLC19A1	pro	YES
SMNDC1	pro	YES
SOD1	both	YES
SPN	pro	YES
SST	pro	YES
SSTR3	pro	YES
STK17A	pro	YES
STK17B	pro	YES
STK3	pro	YES
STK4	pro	YES
TBX5	pro	YES
TGFB2	pro	YES
TIA1	pro	YES

TIAL1	pro	YES
TIMP3	pro	YES
TLR2	pro	YES
TNF	both	YES
TNFRSF10B	pro	YES
TNFRSF25	pro	YES
TNFRSF8	pro	YES
TNFRSF9	pro	YES
TNFSF10	pro	YES
TNFSF14	pro	YES
TNFSF8	pro	YES
TOP2A	pro	YES
TP53	pro	YES
TP53BP2	pro	YES
TP53I3	pro	YES
TP73	pro	YES
TP73L	pro	NO
TPD52L1	pro	YES
TRADD	pro	YES
TRAF3	pro	YES
TRAIP	pro	YES
TUBB	pro	YES
TUBB2C	pro	YES
UNC13B	pro	YES
UTP11L	pro	YES
ZAK	pro	YES
ZNF443	pro	YES
AATF	anti	YES
ACCN1	anti	YES
ACVR1	anti	YES
AGT	anti	YES
AKT1	anti	YES
ALB	anti	YES
ALOX12	anti	YES
ANGPTL4	anti	YES
ANXA1	anti	YES
ANXA4	anti	YES
ANXA5	anti	YES
API5	anti	YES
APIP	anti	YES
ARHGDIA	anti	YES
ATF5	anti	YES
AVEN	anti	YES
AZU1	anti	YES
BAG1	anti	YES
BAG3	anti	YES
BAG4	anti	YES

BAPX1	anti	NO
BCL2	anti	YES
BCL2A1	anti	YES
BCL2L1	anti	YES
BCL2L10	anti	YES
BCL2L2	anti	YES
BDNF	anti	YES
BECN1	anti	YES
BFAR	anti	YES
BIRC2	anti	YES
BIRC3	anti	YES
BIRC4	anti	NO
BIRC5	anti	YES
BIRC7	anti	YES
BNIP2	anti	YES
BRAF	anti	NO
BRE	anti	YES
CASP2	anti	YES
CBX4	anti	YES
CCL2	anti	YES
CD40LG	anti	YES
CD74	anti	YES
CDC2	anti	YES
CFDP1	anti	YES
CFL1	anti	YES
CIAPIN1	anti	YES
CLCF1	anti	YES
CLN3	anti	YES
CRYAA	anti	YES
CRYAB	anti	YES
CSF2	anti	YES
DAD1	anti	YES
DDAH2	anti	YES
DHCR24	anti	YES
EEF1A2	anti	YES
ERC1	anti	YES
FAIM	anti	YES
FAIM2	anti	YES
FAIM3	anti	YES
FCER1G	anti	YES
FGF4	anti	YES
FOXO1	anti	YES
GCLC	anti	YES
GCLM	anti	YES
GDNF	anti	YES
GLO1	anti	YES
GSK3B	anti	YES

GSTP1	anti	YES
HBXIP	anti	YES
HDAC1	anti	YES
HDAC3	anti	YES
HIPK3	anti	YES
HMGB1	anti	YES
HSP90B1	anti	YES
HSPA1A	anti	NO
HSPA1B	anti	NO
HSPA9	anti	YES
HSPB1	anti	YES
IER3	anti	YES
IFI6	anti	YES
IGF1R	anti	YES
IL10	anti	YES
IL1A	anti	YES
IL2	anti	YES
IL2RB	anti	YES
IL3	anti	YES
IL4	anti	YES
IL6	anti	YES
IL7	anti	YES
KRT18	anti	YES
LIG4	anti	YES
MALT1	anti	YES
MAPK8	anti	YES
MAPK8IP2	anti	YES
MCL1	anti	YES
MIF	anti	NO
MKL1	anti	YES
MPO	anti	YES
MSH2	anti	YES
MTL5	anti	YES
MYBL2	anti	YES
NFKB1	anti	YES
NME1	anti	NO
NME5	anti	YES
NME6	anti	YES
NOL3	anti	YES
NPM1	anti	YES
NRG2	anti	YES
NTF3	anti	YES
NUP62	anti	YES
OPA1	anti	YES
PAK7	anti	YES
PAX7	anti	YES
PEA15	anti	YES

PIK3CA	anti	YES
PIK3CG	anti	YES
PIK3R2	anti	YES
PIM1	anti	YES
PIM2	anti	YES
POGK	anti	YES
POLB	anti	YES
PPT1	anti	YES
PRDX2	anti	YES
PRKAA1	anti	YES
PRKCZ	anti	YES
PRLR	anti	YES
PROC	anti	YES
PROP1	anti	YES
PSEN1	anti	YES
RASA1	anti	YES
RBKS	anti	YES
RELA	anti	YES
ROCK1	anti	YES
RTN4	anti	YES
SCG2	anti	YES
SEMA4D	anti	YES
SERPINB2	anti	YES
SERPINB9	anti	YES
SFRP1	anti	YES
SH3GLB1	anti	YES
SMO	anti	YES
SNCA	anti	YES
SOCS2	anti	YES
SOCS3	anti	YES
SON	anti	YES
SPHK1	anti	YES
SPHK2	anti	YES
SPP1	anti	YES
STAMBP	anti	YES
STAT5A	anti	YES
STAT5B	anti	YES
TAX1BP1	anti	YES
TBX3	anti	YES
TEGT	anti	NO
TGFB1	anti	YES
TNFAIP3	anti	YES
TNFAIP8	anti	YES
TNFRSF10D	anti	YES
TNFSF18	anti	YES
TPT1	anti	YES
TRIAP1	anti	YES

TSC22D3	anti	YES
TXNDC1	anti	YES
TXNDC5	anti	YES
VEGFA	anti	YES
VHL	anti	YES
XRCC4	anti	YES
YWHAH	anti	YES
YWHAZ	anti	YES

nes have probe sets in CMAP data

Supplementary Table S4: All interactions from interaction component of NCBI (

13 Selected Drug-Responsive Genes	Product of Selected Genes	Interactant	Interactant geneSymbol
BCL2L11	NC_000002.9	NP_963853.1	FOXO3
BCL2L11	NP_006529.1	NP_000624.1	BCL2
BCL2L11	NP_006529.1	NP_612815.1	BCL2L1
BCL2L11	NP_006529.1	NP_004041.1	BCL2L2
BCL2L11	O43521	P31749	AKT1
BCL2L11	O43521	P10415	BCL2
BCL2L11	O43521	Q16548	BCL2A1
BCL2L11	O43521	Q07817	BCL2L1
BCL2L11	O43521	O43521	BCL2L11
BCL2L11	O43521	Q92843	BCL2L2
BCL2L11	O43521	P63167	DYNLL1
BCL2L11	O43521	P53779	MAPK10
BCL2L11	O43521	P45983	MAPK8
BCL2L11	O43521	P45984	MAPK9
BCL2L11	O43521	Q07820	MCL1
BCL2L11	O43521	Ring finger and KH don	MEX3D
BCL2L11	O43521	P21796	VDAC1
BCL2L11	O43521	P31946	YWHAB
BCL2L11	BioGRID:115335	BioGRID:107068	BCL2
BCL2L11	BioGRID:115335	BioGRID:107069	BCL2A1
BCL2L11		NP_612815.1	BCL2L1
BCL2L11	BioGRID:115335	BioGRID:107070	BCL2L1
BCL2L11	BioGRID:115335	BioGRID:107071	BCL2L2
BCL2L11	BioGRID:115335	BioGRID:107160	BTK
BCL2L11	BioGRID:115335	BioGRID:114206	DYNLL1
BCL2L11	BioGRID:115335	BioGRID:126680	DYNLL2
BCL2L11	BioGRID:115335	BioGRID:109224	GTF2I
BCL2L11	BioGRID:115335	BioGRID:110338	MCL1
BCL2L11	BioGRID:115335	BioGRID:112482	SMARCB1
BCL2L11	BioGRID:115335	BioGRID:113164	UBC
BCL2L11	BioGRID:115335	BioGRID:113259	VDAC1
BCL2L11	BioGRID:115335	BioGRID:249746	Vdac1
BNIP3L	NT_023666.17	NP_004371.1	CREBBP
BNIP3L	NT_023666.17	NP_000537.2	TP53
BNIP3L	O60238	P10415	BCL2
BNIP3L	O60238	Q07817	BCL2L1
BNIP3L	O60238	Q12983	BNIP3
BNIP3L	O60238	O60238	BNIP3L
BNIP3L	O60238	Q9P104	DOK5
BNIP3L	O60238	Q01844	EWSR1
BNIP3L	O60238	Q6NUQ1	RINT1
BNIP3L	O60238	Dudulin 2	STEAP3
BNIP3L	O60238	P17152	TMEM11
BNIP3L	BioGRID:107133	BioGRID:107068	BCL2

BNIP3L	BioGRID:107133	BioGRID:107132	BNIP3
BNIP3L	BioGRID:107133	BioGRID:107133	BNIP3L
BNIP3L	BioGRID:107133	BioGRID:120926	DOK5
BNIP3L	BioGRID:107133	BioGRID:108431	EWSR1
BNIP3L	BioGRID:107133	BioGRID:120533	STEAP3
BNIP3L	BioGRID:107133	BioGRID:114361	TMEM11
MSH6	NP_000170.1	NP_009227.1	BRCA1
MSH6	NP_000170.1	NP_000242.1	MSH2
MSH6	NP_000170.1	NP_000242.1	MSH2
MSH6	NP_000170.1	NP_036354.1	MUTYH
MSH6	NP_000170.1	NP_002583.1	PCNA
MSH6	P52701	Q92793	CREBBP
MSH6	P52701	Q09472	EP300
MSH6	P52701	P43246	MSH2
MSH6	P52701	Q9UIF7	MUTYH
MSH6	P52701	P12004	PCNA
MSH6	BioGRID:109211	BioGRID:106823	AIRE
MSH6	BioGRID:109211	BioGRID:106962	ATM
MSH6	BioGRID:109211	BioGRID:107027	ATR
MSH6	BioGRID:109211	BioGRID:107056	BARD1
MSH6	BioGRID:109211	BioGRID:107110	BLM
MSH6	BioGRID:109211	BioGRID:107140	BRCA1
MSH6	BioGRID:109211	BioGRID:107287	CASP4
MSH6	BioGRID:109211	BioGRID:107536	CHEK1
MSH6	BioGRID:109211	BioGRID:122015	CLSPN
MSH6	BioGRID:109211	BioGRID:108403	ESR1
MSH6	BioGRID:109211	BioGRID:110438	MLH1
MSH6	BioGRID:109211	BioGRID:116134	MORF4L1
MSH6	BioGRID:109211	BioGRID:110501	MRE11A
MSH6	BioGRID:109211	BioGRID:110573	MSH2
MSH6	BioGRID:109211	BioGRID:110694	MYC
MSH6	BioGRID:109211	BioGRID:110763	NBN
MSH6	BioGRID:109211	BioGRID:111142	PCNA
MSH6	BioGRID:109211	BioGRID:111404	PMS2
MSH6	BioGRID:109211	BioGRID:111576	PRKCZ
MSH6	BioGRID:109211	BioGRID:115417	RAD50
MSH6	BioGRID:109211	BioGRID:111913	RFC1
MSH6	BioGRID:109211	BioGRID:113871	SMC1A
MSH6	BioGRID:109211	BioGRID:116256	TOPBP1
MSH6	BioGRID:109211	BioGRID:113164	UBC
NUP62	NP_036478.2	AAC34298.1	DDX3X
NUP62	P37198	Q9Y3C0	CCDC53
NUP62	P37198	O00571	DDX3X
NUP62	P37198	O60941	DTNB
NUP62	P37198	Q9H8Y8	GORASP2
NUP62	P37198	P29084	GTF2E2
NUP62	P37198	Q03933	HSF2
NUP62	P37198	Q13123	IK
NUP62	P37198	O00410	IPO5
NUP62	P37198	Q14974	KPNB1

NUP62	P37198	P49790	NUP153
NUP62	P37198	Q7Z3B4	NUP54
NUP62	P37198	P37198	NUP62
NUP62	P37198	P52948	NUP98
NUP62	P37198	Q9BVL2	NUPL1
NUP62	P37198	P61970	NUTF2
NUP62	P37198	Q9UBU9	NXF1
NUP62	P37198	Q9GZY0	NXF2
NUP62	P37198	P40425	PBX2
NUP62	P37198	P06454	PTMA
NUP62	P37198	P49792	RANBP2
NUP62	P37198	Q9NVV9	THAP1
NUP62	P37198	Q13114	TRAF3
NUP62	P37198	O14980	XPO1
NUP62	P37198	Q96QU8	XPO6
NUP62	BioGRID:117165	BioGRID:119225	CCDC53
NUP62	BioGRID:117165	BioGRID:108171	DTNB
NUP62	BioGRID:117165	BioGRID:115448	G3BP1
NUP62	BioGRID:117165	BioGRID:115237	G3BP2
NUP62	BioGRID:117165	BioGRID:117479	GORASP2
NUP62	BioGRID:117165	BioGRID:109531	HSF2
NUP62	BioGRID:117165	BioGRID:109766	IK
NUP62	BioGRID:117165	BioGRID:110034	KPNA1
NUP62	BioGRID:117165	BioGRID:110035	KPNB1
NUP62	BioGRID:117165	BioGRID:110865	NFX1
NUP62	BioGRID:117165	BioGRID:119759	NUP54
NUP62	BioGRID:117165	BioGRID:115499	NUTF2
NUP62	BioGRID:117165	BioGRID:121026	NXF2
NUP62	BioGRID:117165	BioGRID:114049	OGT
NUP62	BioGRID:117165	BioGRID:114054	RAE1
NUP62	BioGRID:117165	BioGRID:120448	THAP1
NUP62	BioGRID:117165	BioGRID:113039	TRAF3
NUP62	BioGRID:117165	BioGRID:113348	XPO1
NUP62	BioGRID:117165	BioGRID:116821	XPO6
PMAIP1	NC_000018.8	NP_002458.1	MYC
PMAIP1	NC_000018.8	NP_003434.1	ZBTB17
PMAIP1	NP_066950.1	NP_004040.1	BCL2A1
PMAIP1	Q13794	Q07814	BAX
PMAIP1	Q13794	P10415	BCL2
PMAIP1	Q13794	Q16548	BCL2A1
PMAIP1	Q13794	Q07817	BCL2L1
PMAIP1	Q13794	Q07820	MCL1
PMAIP1	Q13794	P02775	PPBP
PMAIP1	Q13794	Q05516	ZBTB16
PMAIP1	BioGRID:111379	BioGRID:107057	BAX
PMAIP1	BioGRID:111379	BioGRID:107068	BCL2
PMAIP1	BioGRID:111379	BioGRID:107069	BCL2A1
PMAIP1	BioGRID:111379	BioGRID:107070	BCL2L1
PMAIP1	BioGRID:111379	BioGRID:107071	BCL2L2
PMAIP1	BioGRID:111379	BioGRID:110338	MCL1

PMAIP1	BioGRID:111379	BioGRID:113498	ZBTB16
SERINC3	BioGRID:116155	BioGRID:116145	KDELR1
SERINC3	BioGRID:116155	BioGRID:116204	KDELR2
SERINC3	BioGRID:116155	BioGRID:116459	PHB2
SERINC3	BioGRID:116155	BioGRID:119570	PTPLAD1
SERINC3	BioGRID:116155	BioGRID:112454	SLC16A1
SERINC3	BioGRID:116155	BioGRID:112703	SURF4
SERINC3	BioGRID:116155	BioGRID:113260	VDAC2
SERINC3	BioGRID:116155	BioGRID:121574	XPO5
SON	P18583	O75400	PRPF40A
SON	P18583	Q8TAD8	SNIP1
SON	P18583	Q15654	TRIP6
SON	P18583	P31946	YWHAB
SON	P18583	P61981	YWHAG
SON	BioGRID:112534	BioGRID:113364	YWHAG
TAX1BP1	NP_006015.4	NP_821074.1	STARD13
TAX1BP1	Q86VP1	Q07960	ARHGAP1
TAX1BP1	Q86VP1	Q9Y3C5	RNF11
TAX1BP1	Q86VP1	Q9Y3M8	STARD13
TAX1BP1	Q86VP1	Q86VP1	TAX1BP1
TAX1BP1	Q86VP1	Q9HA65	TBC1D17
TAX1BP1	Q86VP1	P21580	TNFAIP3
TAX1BP1	Q86VP1	Q9Y4K3	TRAF6
TAX1BP1	BioGRID:114405	BioGRID:114274	RIPK1
TAX1BP1	BioGRID:114405	BioGRID:117941	RNF11
TAX1BP1	BioGRID:114405	BioGRID:124744	STARD13
TAX1BP1	BioGRID:114405	BioGRID:122849	TBC1D17
TAX1BP1	BioGRID:114405	BioGRID:112983	TNFAIP3
TAX1BP1	BioGRID:114405	BioGRID:113041	TRAF6
TNFAIP3	NC_000006.9	Q92750	TAF4B
TNFAIP3	NP_006281.1	NP_003795.1	RIPK1
TNFAIP3	NP_006281.1		TICAM1
TNFAIP3	NP_006281.1	NP_005998.1	ZFAND5
TNFAIP3	P21580	O15111	CHUK
TNFAIP3	P21580	O14920	IKBKB
TNFAIP3	P21580	Q9Y6K9	IKBKG
TNFAIP3	P21580	Q13571	LAPTM5
TNFAIP3	P21580	Q13546	RIPK1
TNFAIP3	P21580	Q9Y3C5	RNF11
TNFAIP3	P21580	Q86VP1	TAX1BP1
TNFAIP3	P21580	Toll-like receptor adapt	TICAM1
TNFAIP3	P21580	P21580	TNFAIP3
TNFAIP3	P21580	Q15025	TNIP1
TNFAIP3	P21580	TNFAIP3 interacting pr	TNIP2
TNFAIP3	P21580	Q13077	TRAF1
TNFAIP3	P21580	Q12933	TRAF2
TNFAIP3	P21580	Q9Y4K3	TRAF6
TNFAIP3	P21580	P31946	YWHAB
TNFAIP3	P21580	P62258	YWHAE
TNFAIP3	P21580	P61981	YWHAG

TNFAIP3	P21580	Q04917	YWHAH
TNFAIP3	P21580	P63104	YWHAZ
TNFAIP3	P21580	O76080	ZFAND5
TNFAIP3	BioGRID:112983	BioGRID:106725	ALDH9A1
TNFAIP3	BioGRID:112983	BioGRID:107291	CASP8
TNFAIP3	BioGRID:112983	BioGRID:107569	CHUK
TNFAIP3	BioGRID:112983	BioGRID:116534	CNKSR2
TNFAIP3	BioGRID:112983	BioGRID:117657	FBXO3
TNFAIP3	BioGRID:112983	BioGRID:108993	GLDC
TNFAIP3	BioGRID:112983	BioGRID:109767	IKBKB
TNFAIP3	BioGRID:112983	BioGRID:114089	IKBKG
TNFAIP3	BioGRID:112983	BioGRID:110030	KIF11
TNFAIP3	BioGRID:112983	BioGRID:110110	LAMP1
TNFAIP3	BioGRID:112983	BioGRID:121540	LRRC47
TNFAIP3	BioGRID:112983	BioGRID:110343	MCM6
TNFAIP3	BioGRID:112983	BioGRID:110799	NDUFS1
TNFAIP3	BioGRID:112983	BioGRID:111511	PPP2R1B
TNFAIP3	BioGRID:112983	BioGRID:120579	PPP6R3
TNFAIP3	BioGRID:112983	BioGRID:111855	RARRES3
TNFAIP3	BioGRID:112983	BioGRID:114274	RIPK1
TNFAIP3	BioGRID:112983	BioGRID:117941	RNF11
TNFAIP3	BioGRID:112983	BioGRID:111977	RNH1
TNFAIP3	BioGRID:112983	BioGRID:114405	TAX1BP1
TNFAIP3	BioGRID:112983	BioGRID:118878	TBK1
TNFAIP3	BioGRID:112983	BioGRID:112983	TNFAIP3
TNFAIP3	BioGRID:112983	BioGRID:115602	TNIP1
TNFAIP3	BioGRID:112983	BioGRID:113037	TRAF1
TNFAIP3	BioGRID:112983	BioGRID:113038	TRAF2
TNFAIP3	BioGRID:112983	BioGRID:113041	TRAF6
TNFAIP3	BioGRID:112983	BioGRID:113361	YWHAB
TNFAIP3	BioGRID:112983	BioGRID:113363	YWHAE
TNFAIP3	BioGRID:112983	BioGRID:113364	YWHAG
TNFAIP3	BioGRID:112983	BioGRID:113365	YWHAH
TNFAIP3	BioGRID:112983	BioGRID:113366	YWHAZ
TUBB	P07437	P04075	ALDOA
TUBB	P07437	Q9NVJ2	ARL8B
TUBB	P07437	P15291	B4GALT1
TUBB	P07437	Q13873	BMPR2
TUBB	P07437	P38398	BRCA1
TUBB	P07437	Q9NV56	C20orf20
TUBB	P07437	Postsynaptic protein CI	CRIPT
TUBB	P07437	Q16555	DPYSL2
TUBB	P07437	P63167	DYNLL1
TUBB	P07437	P03372	ESR1
TUBB	P07437	P63244	GNB2L1
TUBB	P07437	Q13255	GRM1
TUBB	P07437	Q9UBN7	HDAC6
TUBB	P07437	P42858	HTT
TUBB	P07437	P78559	MAP1A
TUBB	P07437	Q02779	MAP3K10

TUBB	P07437	P27816	MAP4
TUBB	P07437	Q6P3T0	MAP6
TUBB	P07437	P10636	MAPT
TUBB	P07437	Q96L34	MARK4
TUBB	P07437	P20591	MX1
TUBB	P07437	P61601	NCALD
TUBB	P07437	O15259	NPHP1
TUBB	P07437	O60260	PARK2
TUBB	P07437	Q9NQP4	PFDN4
TUBB	P07437	P53350	PLK1
TUBB	P07437	Q92930	RAB8B
TUBB	P07437	Q9NS23	RASSF1
TUBB	P07437	O75056	SDC3
TUBB	P07437	O95793	STAU1
TUBB	P07437	Q86SS6	SYT9
TUBB	P07437	Q9UL54	TAOK2
TUBB	P07437	Q9BTW9	TBCD
TUBB	P07437	P21980	TGM2
TUBB	P07437	Q5TCY1	TTBK1
TUBB	P07437	P33981	TTK
TUBB	P07437	Q71U36	TUBA1A
TUBB	P07437	P15498	VAV1
TUBB	P07437	P31946	YWHAB
TUBB	P07437	P61981	YWHAG
TUBB	P07437	P63104	YWHAZ
TUBB	P07437	P43403	ZAP70
TUBB	BioGRID:128444	BioGRID:120503	ARL8B
TUBB	BioGRID:128444	BioGRID:106922	ASCL2
TUBB	BioGRID:128444	BioGRID:116686	FNBP1
TUBB	BioGRID:128444	BioGRID:114593	HGS
TUBB	BioGRID:128444	BioGRID:121760	MARK4
TUBB	BioGRID:128444	BioGRID:116691	NCOA6
TUBB	BioGRID:128444	BioGRID:110927	NPHP1
TUBB	BioGRID:128444	BioGRID:111425	POLH
TUBB	BioGRID:128444	BioGRID:114513	PSTPIP1
TUBB	BioGRID:128444	BioGRID:119719	RAB8B
TUBB	BioGRID:128444	BioGRID:116356	RASSF1
TUBB	BioGRID:128444	BioGRID:111864	RBBP5
TUBB	BioGRID:128444	BioGRID:115027	SDC3
TUBB	BioGRID:128444	BioGRID:116593	SIRT2
TUBB	BioGRID:128444	BioGRID:114574	SMC3
TUBB	BioGRID:128444	BioGRID:112497	SUMO2
TUBB	BioGRID:128444	BioGRID:126802	SYT9
VEGFA	AF095785.1	NP_001521.1	HIF1A
VEGFA	AF095785.1	NP_000312.1	RB1
VEGFA	NP_003367.3	AAD48080.1	ADAMTS1
VEGFA	NP_003367.3	NP_002010.1	FLT1
VEGFA	NP_003367.3	NP_002010.1	FLT1
VEGFA	NP_003367.3	NP_002244.1	KDR
VEGFA	NP_003367.3	NP_002244.1	KDR

VEGFA	NP_003367.3	NP_002244.1	KDR
VEGFA	NP_003367.3	NP_003864.2	NRP1
VEGFA	NP_003367.3	NP_003864.2	NRP1
VEGFA	NP_003367.3	NP_957718.1	NRP2
VEGFA	NP_003367.3		NRP2
VEGFA	NP_003367.3		
VEGFA	P15692	Q9UHI8	ADAMTS1
VEGFA	P15692	P31749	AKT1
VEGFA	P15692	P16989	CSDA
VEGFA	P15692	P29279	CTGF
VEGFA	P15692	P29323	EPHB2
VEGFA	P15692	P17948	FLT1
VEGFA	P15692	P35052	GPC1
VEGFA	P15692	Q16270	IGFBP7
VEGFA	P15692	P35968	KDR
VEGFA	P15692	O14786	NRP1
VEGFA	P15692	O60462	NRP2
VEGFA	P15692	P49763	PGF
VEGFA	P15692	Q13275	SEMA3F
VEGFA	P15692	P09486	SPARC
VEGFA	P15692	P22105	TNXB
VEGFA	P15692	P15692	VEGFA
VEGFA	P15692	P49765	VEGFB
VEGFA	P15692	P04004	VTN
VEGFA	P15692	P67809	YBX1
VEGFA	BioGRID:113265	BioGRID:114888	ADAMTS1
VEGFA	BioGRID:113265	BioGRID:107872	CTGF
VEGFA	BioGRID:113265	BioGRID:109079	GPC1
VEGFA	BioGRID:113265	BioGRID:109552	HSP90AA1
VEGFA	BioGRID:113265	BioGRID:109540	HSPA4
VEGFA	BioGRID:113265	BioGRID:109711	IGFBP7
VEGFA	BioGRID:113265	BioGRID:109992	KDR
VEGFA	BioGRID:113265	BioGRID:114356	NRP1
VEGFA	BioGRID:113265	BioGRID:111249	PGF
VEGFA	BioGRID:113265	BioGRID:112560	SPARC
VEGFA	BioGRID:113265	BioGRID:113265	VEGFA
VEGFA	BioGRID:113265	BioGRID:113287	VTN
NOL3	NP_003937.1	NP_620116.1	BAX
NOL3	NP_003937.1		CASP8
NOL3	NP_003937.1	NP_003815.1	FADD
NOL3	NP_003937.1		FAS
NOL3	NP_003937.1	NP_003937.1	NOL3
NOL3	O60936	Q07814	BAX
NOL3	O60936	P42575	CASP2
NOL3	O60936	Q14790	CASP8
NOL3	O60936	P68400	CSNK2A1
NOL3	O60936	Q9NVF9	ETNK2
NOL3	O60936	Q13158	FADD
NOL3	O60936	Q9GZT8	NIF3L1

NOL3	O60936	O60936	NOL3
NOL3	O60936	P78324	SIRPA
NOL3	O60936	Q13242	SRSF9
NOL3	O60936	P0C1Z6	TFPT
NOL3	O60936	P50552	VASP
NOL3	BioGRID:114477	BioGRID:107285	CASP2
NOL3	BioGRID:114477	BioGRID:107291	CASP8
NOL3	BioGRID:114477	BioGRID:114364	CFLAR
NOL3	BioGRID:114477	BioGRID:120519	ETNK2
NOL3	BioGRID:114477	BioGRID:114302	FADD
NOL3	BioGRID:114477	BioGRID:106851	FAS
NOL3	BioGRID:114477	BioGRID:121922	NIF3L1
NOL3	BioGRID:114477	BioGRID:114477	NOL3
NOL3	BioGRID:114477	BioGRID:118896	PACSIN3
NOL3	BioGRID:114477	BioGRID:116440	PNKP
NOL3	BioGRID:114477	BioGRID:126752	SIRPA
NOL3	BioGRID:114477	BioGRID:114231	SRSF9
NOL3	BioGRID:114477	BioGRID:112986	TNFRSF1A
NOL3	BioGRID:114477	BioGRID:114258	TNFRSF25
NOL3	BioGRID:114477	BioGRID:114257	TRADD
NOL3	BioGRID:114477	BioGRID:113251	VASP
NOL3	BioGRID:114477	BioGRID:43363	ced-3

Gene Database for 13 selected candidate apoptotic genes

Interaction Source	Reference	Description of Interaction
BIND	PubMed	FoxO3a interacts with the BCL2L11 (Bim) promoter.
BIND	PubMed	Bim interacts with Bcl-2.
BIND	PubMed	Bim interacts with Bcl-XL.
BIND	PubMed	Bim interacts with Bcl-w.
HPRD	PubMed	
HPRD	PubMed	
HPRD	PubMed	
HPRD	PubMed	
HPRD	PubMed	
HPRD	PubMed	
HPRD	PubMed	
HPRD	PubMed	
HPRD	PubMed	
HPRD	PubMed	
HPRD	PubMed	
HPRD	PubMed	
HPRD	PubMed	
HPRD	PubMed	
BioGRID	PubMed	Affinity Capture-Western; Reconstituted Complex; Tw
BioGRID	PubMed	Two-hybrid
BIND	PubMed	Bcl-XL interacts with Bim. This interaction was modell
BioGRID	PubMed	Affinity Capture-Western; FRET; Two-hybrid
BioGRID	PubMed	Affinity Capture-Western; Two-hybrid
BioGRID	PubMed	Reconstituted Complex
BioGRID	PubMed	Reconstituted Complex
BioGRID	PubMed	Reconstituted Complex
BioGRID	PubMed	Reconstituted Complex
BioGRID	PubMed	Affinity Capture-Western; Two-hybrid
BioGRID	PubMed	Reconstituted Complex
BioGRID	PubMed	Affinity Capture-Western
BioGRID	PubMed	Affinity Capture-Western
BioGRID	PubMed	Reconstituted Complex
BIND	PubMed	CBP interacts with Bnip3L.
BIND	PubMed	p53 interacts with Bnip3L.
HPRD	PubMed	
HPRD	PubMed	
HPRD	PubMed	
HPRD	PubMed	
HPRD	PubMed	
HPRD	PubMed	
HPRD	PubMed	
HPRD	PubMed	
HPRD	PubMed	
BioGRID	PubMed	Affinity Capture-Western

BioGRID	PubMed	Two-hybrid
BioGRID	PubMed	Two-hybrid
BioGRID	PubMed	Two-hybrid
BioGRID	PubMed	Two-hybrid
BioGRID	PubMed	Affinity Capture-Western; Two-hybrid
BioGRID	PubMed	Two-hybrid
BIND	PubMed	BRCA1 associates with MSH6.
BIND	PubMed	MSH2 interacts with MSH6.
BIND	PubMed	MSH2 interacts with MSH6.
BIND	PubMed	hMYH interacts with hMSH6.
BIND	PubMed	MSH6 is a PCNA-binding protein
HPRD	PubMed	
HPRD	PubMed	
HPRD	PubMed	
HPRD	PubMed	
HPRD	PubMed	
BioGRID	PubMed	Affinity Capture-MS
BioGRID	PubMed	Co-purification
BioGRID	PubMed	Affinity Capture-Western
BioGRID	PubMed	Reconstituted Complex
BioGRID	PubMed	Co-purification
BioGRID	PubMed	Affinity Capture-MS; Co-purification; Reconstituted Co
BioGRID	PubMed	Affinity Capture-MS
BioGRID	PubMed	Affinity Capture-Western
BioGRID	PubMed	Affinity Capture-Western
BioGRID	PubMed	Affinity Capture-MS
BioGRID	PubMed	Co-purification
BioGRID	PubMed	Affinity Capture-MS
BioGRID	PubMed	Co-purification
BioGRID	PubMed	Affinity Capture-MS; Co-purification; Reconstituted Co
BioGRID	PubMed	Affinity Capture-MS
BioGRID	PubMed	Co-purification
BioGRID	PubMed	Affinity Capture-MS; Affinity Capture-Western; Far W
BioGRID	PubMed	Affinity Capture-MS
BioGRID	PubMed	Affinity Capture-Western
BioGRID	PubMed	Co-purification
BioGRID	PubMed	Co-purification
BioGRID	PubMed	Reconstituted Complex
BioGRID	PubMed	Affinity Capture-Western
BioGRID	PubMed	Affinity Capture-Western
BIND	PubMed	DDX3 interacts with Nup62.
HPRD	PubMed	
HPRD	PubMed	
HPRD	PubMed	
HPRD	PubMed	
HPRD	PubMed	
HPRD	PubMed	
HPRD	PubMed	
HPRD	PubMed	
HPRD	PubMed	

HPRD	PubMed	
HPRD	PubMed	
HPRD	PubMed	
HPRD	PubMed	
HPRD	PubMed	
HPRD	PubMed	
HPRD	PubMed	
HPRD	PubMed	
HPRD	PubMed	
HPRD	PubMed	
HPRD	PubMed	
HPRD	PubMed	
HPRD	PubMed	
HPRD	PubMed	
HPRD	PubMed	
BioGRID	PubMed	Two-hybrid
BioGRID	PubMed	Two-hybrid
BioGRID	PubMed	Affinity Capture-MS
BioGRID	PubMed	Affinity Capture-MS
BioGRID	PubMed	Two-hybrid
BioGRID	PubMed	Reconstituted Complex; Two-hybrid
BioGRID	PubMed	Two-hybrid
BioGRID	PubMed	Reconstituted Complex
BioGRID	PubMed	Affinity Capture-Western; Reconstituted Complex
BioGRID	PubMed	Reconstituted Complex
BioGRID	PubMed	Two-hybrid
BioGRID	PubMed	Reconstituted Complex
BioGRID	PubMed	Reconstituted Complex
BioGRID	PubMed	Biochemical Activity
BioGRID	PubMed	Affinity Capture-MS
BioGRID	PubMed	Two-hybrid
BioGRID	PubMed	Affinity Capture-Western; Two-hybrid
BioGRID	PubMed	Affinity Capture-Western; Reconstituted Complex
BioGRID	PubMed	Two-hybrid
BIND	PubMed	c-Myc interacts with NOXA promoter.
BIND	PubMed	Miz1 interacts with NOXA promoter.
BIND	PubMed	Noxa interacts with A1. This interaction was modelled
HPRD	PubMed	
HPRD	PubMed	
HPRD	PubMed	
HPRD	PubMed	
HPRD	PubMed	
HPRD	PubMed	
HPRD	PubMed	
BioGRID	PubMed	Affinity Capture-Western
BioGRID	PubMed	Affinity Capture-Western; Protein-peptide
BioGRID	PubMed	Protein-peptide
BioGRID	PubMed	Affinity Capture-Western; Protein-peptide
BioGRID	PubMed	Protein-peptide
BioGRID	PubMed	Affinity Capture-Western

BioGRID	PubMed	Two-hybrid
BioGRID	PubMed	Affinity Capture-MS
BioGRID	PubMed	Affinity Capture-MS
BioGRID	PubMed	Affinity Capture-MS
BioGRID	PubMed	Affinity Capture-MS
BioGRID	PubMed	Affinity Capture-MS
BioGRID	PubMed	Affinity Capture-MS
BioGRID	PubMed	Affinity Capture-MS
BioGRID	PubMed	Affinity Capture-MS
HPRD	PubMed	
HPRD	PubMed	
HPRD	PubMed	
HPRD	PubMed	
HPRD	PubMed	
BioGRID	PubMed	Affinity Capture-MS
BIND	PubMed	STARD13 (RhoGAP) interacts with TAX1BP1 (Tax1B
HPRD	PubMed	
HPRD	PubMed	
HPRD	PubMed	
HPRD	PubMed	
HPRD	PubMed	
HPRD	PubMed	
HPRD	PubMed	
BioGRID	PubMed	Affinity Capture-Western
BioGRID	PubMed	Affinity Capture-Western
BioGRID	PubMed	Two-hybrid
BioGRID	PubMed	Two-hybrid
BioGRID	PubMed	Affinity Capture-Western; Two-hybrid
BioGRID	PubMed	Affinity Capture-Western; Two-hybrid
BIND	PubMed	TAF4B (hTAF4b) interacts with TNFAIP3 (A20) prom
BIND	PubMed	A20 ubiquitinates and de-ubiquitinates RIP.
BIND	PubMed	An unspecified isoform of TRIF interacts with A20. Th
BIND	PubMed	ZNF216 interacts with A20. This interaction was mod
HPRD	PubMed	
HPRD	PubMed	
HPRD	PubMed	
HPRD	PubMed	
HPRD	PubMed	
HPRD	PubMed	
HPRD	PubMed	
HPRD	PubMed	
HPRD	PubMed	
HPRD	PubMed	
HPRD	PubMed	
HPRD	PubMed	
HPRD	PubMed	
HPRD	PubMed	
HPRD	PubMed	
HPRD	PubMed	
HPRD	PubMed	

HPRD	PubMed	
HPRD	PubMed	
HPRD	PubMed	
BioGRID	PubMed	Affinity Capture-MS
BioGRID	PubMed	Affinity Capture-Western
BioGRID	PubMed	Affinity Capture-Western
BioGRID	PubMed	Affinity Capture-MS
BioGRID	PubMed	Affinity Capture-MS
BioGRID	PubMed	Affinity Capture-MS
BioGRID	PubMed	Biochemical Activity
BioGRID	PubMed	Affinity Capture-Western; Two-hybrid
BioGRID	PubMed	Affinity Capture-MS
BioGRID	PubMed	Co-fractionation
BioGRID	PubMed	Affinity Capture-MS
BioGRID	PubMed	Affinity Capture-MS
BioGRID	PubMed	Affinity Capture-MS
BioGRID	PubMed	Affinity Capture-MS
BioGRID	PubMed	Affinity Capture-MS
BioGRID	PubMed	Affinity Capture-Western
BioGRID	PubMed	Affinity Capture-Western; Biochemical Activity
BioGRID	PubMed	Affinity Capture-Western
BioGRID	PubMed	Affinity Capture-MS
BioGRID	PubMed	Affinity Capture-Western; Two-hybrid
BioGRID	PubMed	Affinity Capture-MS
BioGRID	PubMed	Two-hybrid
BioGRID	PubMed	Affinity Capture-Western; Two-hybrid
BioGRID	PubMed	Affinity Capture-Western; Two-hybrid
BioGRID	PubMed	Affinity Capture-Western; Two-hybrid
BioGRID	PubMed	Affinity Capture-Western
BioGRID	PubMed	Affinity Capture-MS; Affinity Capture-Western; Recon
BioGRID	PubMed	Affinity Capture-Western
BioGRID	PubMed	Two-hybrid
BioGRID	PubMed	Affinity Capture-MS; Affinity Capture-Western; Recon
BioGRID	PubMed	Affinity Capture-Western; Reconstituted Complex; Tw
HPRD	PubMed	
HPRD	PubMed	
HPRD	PubMed	
HPRD	PubMed	
HPRD	PubMed	
HPRD	PubMed	
HPRD	PubMed	
HPRD	PubMed	
HPRD	PubMed	
HPRD	PubMed	
HPRD	PubMed	
HPRD	PubMed	
HPRD	PubMed	
HPRD	PubMed	
HPRD	PubMed	
HPRD	PubMed	

HPRD	PubMed	
HPRD	PubMed	
HPRD	PubMed	
HPRD	PubMed	
HPRD	PubMed	
HPRD	PubMed	
HPRD	PubMed	
HPRD	PubMed	
HPRD	PubMed	
HPRD	PubMed	
HPRD	PubMed	
HPRD	PubMed	
HPRD	PubMed	
HPRD	PubMed	
HPRD	PubMed	
HPRD	PubMed	
HPRD	PubMed	
HPRD	PubMed	
HPRD	PubMed	
HPRD	PubMed	
HPRD	PubMed	
HPRD	PubMed	
HPRD	PubMed	
HPRD	PubMed	
HPRD	PubMed	
HPRD	PubMed	
BioGRID	PubMed	Affinity Capture-Western
BioGRID	PubMed	Affinity Capture-Western
BioGRID	PubMed	Affinity Capture-MS
BioGRID	PubMed	Reconstituted Complex
BioGRID	PubMed	Affinity Capture-MS
BioGRID	PubMed	Affinity Capture-MS; Co-purification
BioGRID	PubMed	Affinity Capture-Western
BioGRID	PubMed	Affinity Capture-MS
BioGRID	PubMed	Affinity Capture-MS
BioGRID	PubMed	Affinity Capture-Western
BioGRID	PubMed	Reconstituted Complex
BioGRID	PubMed	Co-purification
BioGRID	PubMed	Reconstituted Complex
BioGRID	PubMed	Biochemical Activity
BioGRID	PubMed	Affinity Capture-Western
BioGRID	PubMed	Affinity Capture-MS
BioGRID	PubMed	Affinity Capture-Western; Reconstituted Complex
BIND	PubMed	HIF1A (HIF-1-alpha) interacts with the VEGF promote
BIND	PubMed	RB1 (pRB) interacts with the VEGF promoter.
BIND	PubMed	ADAMTS1 interacts with VEGF165
BIND	PubMed	VEGF-A interacts with VEGFR-1. This interaction was
BIND	PubMed	VEGF165 interacts with Flt-1. This interaction was mo
BIND	PubMed	VEGF interacts with KDR.
BIND	PubMed	VEGF-A interacts with VEGFR-2. This interaction was

BIND	PubMed	VEGF165 interacts with KDR. This interaction was mo
BIND	PubMed	VEGF165 interacts with neuropilin-1. This interaction
BIND	PubMed	NRP1 interacts with VEGF165.
BIND	PubMed	NP-2 interacts with VEGF165.
BIND	PubMed	NP-2 interacts with VEGF145.
BIND	PubMed	GFB-204 interacts with VEGF.
HPRD	PubMed	
HPRD	PubMed	
HPRD	PubMed	
HPRD	PubMed	
HPRD	PubMed	
HPRD	PubMed	
HPRD	PubMed	
HPRD	PubMed	
HPRD	PubMed	
HPRD	PubMed	
HPRD	PubMed	
HPRD	PubMed	
HPRD	PubMed	
HPRD	PubMed	
HPRD	PubMed	
HPRD	PubMed	
HPRD	PubMed	
HPRD	PubMed	
HPRD	PubMed	
BioGRID	PubMed	Affinity Capture-Western; Co-purification; Reconstitut
BioGRID	PubMed	Affinity Capture-Western; Reconstituted Complex
BioGRID	PubMed	Reconstituted Complex
BioGRID	PubMed	Affinity Capture-Western
BioGRID	PubMed	Affinity Capture-Western
BioGRID	PubMed	Reconstituted Complex
BioGRID	PubMed	Biochemical Activity
BioGRID	PubMed	Affinity Capture-Western; Reconstituted Complex
BioGRID	PubMed	Co-purification
BioGRID	PubMed	Far Western
BioGRID	PubMed	Co-crystal Structure; Co-purification
BioGRID	PubMed	Reconstituted Complex
BIND	PubMed	ARC interacts with Bax.
BIND	PubMed	ARC interacts with procaspase-8.
BIND	PubMed	ARC interacts with FADD.
BIND	PubMed	ARC interacts with an unspecified isoform of Fas.
BIND	PubMed	ARC homodimerizes via its CARD domain.
HPRD	PubMed	
HPRD	PubMed	
HPRD	PubMed	
HPRD	PubMed	
HPRD	PubMed	
HPRD	PubMed	
HPRD	PubMed	

HPRD	PubMed	
HPRD	PubMed	
HPRD	PubMed	
HPRD	PubMed	
HPRD	PubMed	
BioGRID	PubMed	Affinity Capture-Western
BioGRID	PubMed	Affinity Capture-Western; Phenotypic Suppression
BioGRID	PubMed	Phenotypic Suppression
BioGRID	PubMed	Two-hybrid
BioGRID	PubMed	Phenotypic Suppression
BioGRID	PubMed	Phenotypic Suppression
BioGRID	PubMed	Two-hybrid
BioGRID	PubMed	Affinity Capture-Western; Two-hybrid
BioGRID	PubMed	Affinity Capture-MS
BioGRID	PubMed	Affinity Capture-MS
BioGRID	PubMed	Two-hybrid
BioGRID	PubMed	Affinity Capture-Western; Reconstituted Complex; Two-hybrid
BioGRID	PubMed	Phenotypic Suppression
BioGRID	PubMed	Phenotypic Suppression
BioGRID	PubMed	Phenotypic Suppression
BioGRID	PubMed	Two-hybrid
BioGRID	PubMed	Affinity Capture-Western; Phenotypic Suppression

/o-hybrid

led on a demonstrated interaction between Bcl-XL from an unspecified species and

omplex; Two-hybrid

omplex

estern; Protein-peptide

on a demonstrated interaction between human Noxa and mouse A1.

P).

oter.

is interaction was modeled on a demonstrated interaction between TRIF from an un
eled on a demonstrated interaction between human ZNF216 and A20 from an unspe

stituted Complex; Two-hybrid

stituted Complex; Two-hybrid
vo-hybrid

er.

s modeled on a demonstrated interaction between human VEGF-A and VEGFR-1 fro
odeled on a demonstrated interaction between human VEGF165 and Flt-1 from an u

s modeled on a demonstrated interaction between human VEGF-A and VEGFR-2 fro

odeled on a demonstrated interaction between human VEGF165 and KDR from an u
was modeled on a demonstrated interaction between human VEGF165 and neuropi

ed Complex

human Bim.

specified species and A20 from an unspecified species.
ecified species.

om an unspecified species.
nspecified species.

om an unspecified species.

ınspecified species.
lin-1 from an unspecified species.
